# Irrigation System, Rather than Nitrogen Fertilizer Application, Affects the Quantities of Functional Genes Related to N_2_O Production in Potato Cropping

**DOI:** 10.3390/microorganisms13040741

**Published:** 2025-03-25

**Authors:** Laura Charlotte Storch, Katharina Schulz, Jana Marie Kraft, Annette Prochnow, Liliane Ruess, Benjamin Trost, Susanne Theuerl

**Affiliations:** 1 Leibniz Institute for Agricultural Engineering and Bioeconomy, Max-Eyth-Allee 100, 14469 Potsdam, Germanysusanne.theuerl@googlemail.com (S.T.); 2 Institute of Biology, Ecology Group, Humboldt-Universität zu Berlin, Philippstr. 13, 10115 Berlin, Germanyliliane.ruess@biologie.hu-berlin.de (L.R.); 3 Albrecht Daniel Thaer Institute for Agricultural and Horticultural Sciences, Humboldt Universität zu Berlin, Hinter der Reinhardtstr. 6–8, 10115 Berlin, Germany; 4 Field Research Station Marquardt, Leibniz Institute for Agricultural Engineering and Bioeconomy, 14469 Potsdam, Germany

**Keywords:** nitrous oxide emissions, drip and sprinkler irrigation, fertigation, nitrogen cycle, functional genes, nitrifier denitrification

## Abstract

The spatial and temporal distribution of water and nitrogen supply affects soil-borne nitrous oxide (N_2_O) emissions. In this study, the effects of different irrigation technologies (no irrigation, sprinkler irrigation and drip irrigation) and nitrogen (N) application types (no fertilizer, broadcasted and within irrigation water) on N_2_O flux rates and the quantities of functional genes involved in the N cycle in potato cropping were investigated over an entire season. The volume of irrigation water affected microbial N_2_O production, with the highest N_2_O flux rates found under sprinkler irrigation conditions, followed by drip and no irrigation. Nitrifier denitrification was identified as the potential pre-dominant pathway stimulated by fluctuations in aerobic-anaerobic soil conditions, especially under sprinkler irrigation. Regarding the different N application types, increased N use efficiency under fertigation was expected. However, N_2_O flux rates were not significantly reduced compared to broadcasted N application under drip irrigation. On average, the N_2_O fluxes were higher during the first half of the season, which was accompanied by a low N use efficiency of the potato crops. Potato crops mainly require N at later growth stages. Due to the different water and nutrient demand of potatoes, an adjusted application of fertilizer and water based on crop demand could reduce N_2_O emissions.

## 1. Introduction

Nitrous oxide (N_2_O) is a potent greenhouse gas with a global warming potential 273 times higher than that of carbon dioxide, and its increasing atmospheric concentration is of global concern [1]. These emissions mainly originate from the agricultural sector and are expected to increase, as agricultural crop production needs to be increased to maintain the growing world population [2,3,4,5]. Higher agricultural production is often accompanied by the increased application of mineral fertilizer, in particular nitrogen (N) [2,5,6]. The vast majority of current agricultural systems are characterized by a low nitrogen use efficiency (NUE) or low apparent nitrogen recoveries (ANR), with N losses inter alia through nitrate leaching and/or the generation of N_2_O [5,6,7,8,9,10].

Additionally, irrigation is increasingly important for guaranteeing efficient agricultural production due to progressing climate change [3,11,12]. Irrigation in general increases soil water content and anaerobic soil conditions, resulting in higher N_2_O emissions [3,13,14]. It has been shown that different irrigation systems lead to differences in GHG emissions, with 46% higher N_2_O emissions under sprinkler irrigation compared to water-saving drip irrigation systems [3,15,16,17,18]. Water application under drip irrigation results in decreased soil water content with reduced effects on microbial N_2_O production [3,13]. However, due to the great frequency of wetting and drying cycles next to the dripper, some studies found higher emission rates under drip irrigation [19,20]. Another agricultural management measure with the aim of reducing the availability of N for microbial-mediated processes and hence reducing N_2_O emissions is fertigation, in which dissolved N is applied in multiple small doses through the application of irrigation water, e.g., as in [3,21,22].

N_2_O is mainly generated as a product of microbial-mediated processes like denitrification or as a “by-product” of nitrification, including nitrifier denitrification, or dissimilatory nitrate reduction (NO_3_^−^ to NH_4_^+^) [13,14,23,24,25,26]. These processes are catalyzed by different enzymes encoded by distinct functional genes, e.g., [14,23,24]. Among these functional genes, *amo*A (encoding ammonium monooxygenase), *nxr*B (encoding nitrite oxidoreductase), *nar*G (encoding nitrate reductase), *nir*K/*nir*S (encoding nitrite reductase), and *nos*Z (encoding N_2_O reductase) have been most frequently investigated [13,14,27,28]. These microbial-mediated processes are influenced by various factors, e.g., soil moisture, oxygen availability, temperature, and the availability of reactive N compounds (e.g., ammonium (NH_4_^+^) and nitrate (NO_3_^−^)), as well as root exudates segregated by the cultivated crop, which in turn could influence the microbial N_2_O production potential [13,14,23].

Therefore, it is not only essential to assess the effects of agronomic management measures on N_2_O flux rates, and hence N_2_O emissions, but also to better understand the underlying microbial-mediated mechanisms in order to guarantee sustainable agricultural production with high crop yields and low detrimental environmental impacts.

Even though several authors have investigated soil microbial communities and their related N_2_O production potential in various cropping systems, e.g., [29,30,31,32,33,34], little is known about microbial N_2_O production as influenced by different fertilizer application types and irrigation systems over an entire cropping season. In order to investigate the effects of different fertilization and irrigation strategies on the microbial N_2_O production potential, eight different treatments combining different fertilization application (broadcasted or fertigation) and irrigation types (none, sprinkler, and drip irrigation) were established in potato cropping. The treatments included (1) zero irrigation without N fertilizer (ZI-ZN), (2) zero irrigation with broadcasted N fertilizer (ZI-N), (3) sprinkler irrigation without N (SI-ZN), (4) sprinkler irrigation with N (SI-N), (5) drip irrigation without N (DI-ZN), (6) drip irrigation with N (DI-N), (7) fertigation with N (F), and (8) fertigation without crops (F-ZC). The objective of this study was to elucidate the underlying genetically determined microbial-mediated pathways of N_2_O formation depending on the type, amount, and time of water and fertilizer application.

We addressed the following hypotheses regarding irrigation effects:

- N_2_O flux rates differ between the treatments, with the highest flux rates expected under sprinkler irrigation without broadcasted N application (SI-ZN) due to it having the highest water volume application and thus promoting N_2_O production via the denitrification process, followed by drip irrigation without fertilizer application (DI-ZN), as a reduced water volume is directly applied to the rhizosphere of the crops.
- The lowest N_2_O flux rates are expected under zero irrigation without fertilizer application (ZI-ZN).

We addressed the following hypotheses regarding fertilization effects:

- The application of several small N doses in irrigation water by fertigation (F) will lead to lower N_2_O flux rates compared to the broadcasted N application under sprinkler irrigation (SI-N) due to a better N use efficiency of the potato crops.
- Fertigation will potentially lead to more diverse N_2_O production pathways, whereas the broadcasted N application under drip irrigation leads to intermediate N_2_O flux rates.
- The absence of plants in fertigation without crops (F-ZC) may lead to increased N_2_O emissions due to reduced competition for nitrogen between plants and soil microorganisms.

## 2. Materials and Methods

### 2.1. Experimental Site and N_2_O Flux Measurement

The study was conducted in 2020 at the Field Research Station of the Leibniz Institute for Agricultural Engineering and Bioeconomy in Marquardt (Federal State Brandenburg, Germany; 52°28′02″ N 12°57′37″ E). The average annual temperature in 2020 at the Field Research Station was 11.6 °C with a mean annual precipitation of 407 mm (see Figure S1). The soil is characterized as loamy sand, with 76% sand, 13% clay, and 12% silt, with an organic carbon (C_org_) content of 0.5% and a pH value of 6.75.

Eight different management variants, each in three replicates (plots of 4.5 m × 8 m each), were established in a complete randomized block design. The treatments covered eight combinations of varying temporal and spatial distributions of water and nitrogen supply, which reflected the following current application technologies: zero irrigation with and without (zero) N application (ZI-N, ZI-ZN), sprinkler irrigation with and without (zero) N application (SI-N, SI-ZN), drip irrigation with and without (zero) N application (DI-N, DI-ZN), and fertigation (simultaneous application of water and N fertilizer) with and without (zero) crops (F, F-ZC) (Table 1).

For drip irrigation, Streamline™ X 16080 tubes (NETAFIM™, Tel Aviv, Israel) were installed on the ridges; for sprinkler irrigationZoomMaxx (GARDENA^®^, Ulm, Germany) was installed in the center of the respective plots. The amount of irrigation water has been adapted to the site-specific drip irrigation and fertigation system. Drip-irrigated plots received 37 L m^−2^, fertigated plots 59 L m^−2^, and sprinkler-irrigated plots 120 L m^−2^ of water (Supplementary Table S1). Prior to potato planting, all plots were fertilized once with potassium (250 kg K ha^−1^), magnesium (60 kg Mg ha^−1^), and sulfur (170 kg S ha^−1^) [35] using Patentkali^®^ (K + S AG, Kassel, Germany). Throughout the season, N fertilizer YaraLiva^®^ Tropicote^®^ (Yara International ASA, Oslo, Norway; chemical composition: 15.5% total N, 14.4% nitrate N, 1.1% ammonium N, and 25% water soluble calcium oxide) was applied three times (total amount of N: 150 kg N ha^−1^) in all fertilized plots, except for the fertigated plots, where fertilizer (Yara Liva™ Calcinit™, International ASA, Oslo, Norway; 15.5% total N, 14.4% nitrate N, 1.1% ammonium N, and 26% water soluble calcium oxide) was dissolved in the irrigation water and applied at regular intervals (total amount of N: 151 kg N ha^−1^) (Supplementary Table S1). Agrochemicals in terms of fungicides (Acrobat^®^ Plus WG, BASF SE; Ludwigshafen am Rhein, Germany; 2 kg ha^−1^), insecticides (Biscaya^®^, Bayer AG, Leverkusen, Germany; 0.3 L ha^−1^), and herbicides (Boxer^®^, Syngenta Group Co. Ltd., Shanghai, China; 5 L ha^−1^) were applied according to common practice while all plots were treated equally, including F-ZC. Additionally, all plots were weeded manually on a weekly basis or when necessary. Except for a few weeds, the F-ZC-treated plots exhibited bare soil.

N_2_O measurements started three weeks after the potatoes were planted, covering an entire cropping season of 16 weeks from 26 May to 15 September 2020. The soil-borne N_2_O fluxes were measured weekly using the closed chamber method [28,36]. The chosen weekly basis for gas measurements is in accordance with recommendations from Charteris et al. (2020) [37] and has been applied in several studies, e.g., [28,35,37,38,39].

Briefly, in each plot, one polyvinyl chloride (PVC) collar (L × B × H: 80 × 80 × 65 cm) was permanently installed, covering two potato plants in one row. Considering a ridge width of 75 cm, 93% of the area inside the installed collars was covered by the ridge, and hence 7% by the furrow. During the measurements, the collars were closed gas-tight with a non-transparent PVC chamber (78 × 78 × 52 cm). Gas samples were taken every 20 min for one hour, resulting in four samples per chamber and plot. Gas measurements took place in the mid-morning hours between 9 and 11 a.m. N_2_O flux rates were calculated according to [40] by linear regression using the slope of the temporal change of the N_2_O concentration inside the chamber. Area-related cumulative N_2_O emissions were estimated by accumulation of daily fluxes, which were calculated by linear interpolation of the weekly measured fluxes [35,40,41]. Yield-related N_2_O emissions were estimated by dividing the calculated area-related cumulative N_2_O emissions by the recorded crop yields. For the determination of the crop yield, in each plot, two central undisturbed rows were harvested by hand at the end of season. To avoid an overestimation of the total yield due to edge effects, two potato plants at each end of the row were omitted. Based on the weight of the harvested potatoes per row, excluding the potatoes located at the edge of the row, the crop yield per plot were calculated in tons per hectare (t ha^−1^). During gas sampling, soil temperature was measured every 20 min with a penetration thermometer (Testo SE & Co. KGaA, Titisee-Neustadt, Germany) for the ridge (0–10 cm). For statistical analysis, the mean soil temperature per sampling day was calculated.

### 2.2. Analysis of Mineral Nitrogen and Soil Moisture

For each treatment, five soil samples (0–20 cm soil depth) per plot were taken to analyze the mineral N (Nmin) content (see Supplementary Table S2), including ammonium (NH_4_^+^), nitrate (NO_3_^−^), and nitrite (NO_2_^−^), according to VDLUFA (1991). The determination of volumetric water content (VWC) and bulk density was carried out as described in [28]. From these values, the water-filled pore space (WFPS) was determined according to [42].

### 2.3. Determining Nitrogen Use Efficiency (NUE) and Apparent Nitrogen Recovery (ANR)

Potato samples (tuber) were taken and analyzed (dry matter content, nitrogen content) once a month (June, July, August, and September; one complete plant per plot and sampling date) in order to determine the nitrogen use efficiency (NUE) and apparent nitrogen recovery (ANR). NUE was calculated according to [43]. ANR was calculated according to [7,10].

### 2.4. Functional Profiling of Relevant Genes of the Nitrogen Cycle

For profiling of the functional genes within the N cycle, five distinct sample dates out of the entire vegetation period were analyzed. These dates corresponded to the seasonal development of N_2_O fluxes while simultaneously covering the different crop developmental stages, including the early vegetative period with a strong growth rate, the transition from the vegetative to the flowering period, and the maturation and senescence periods. Per plot, three soil cores at a depth of 0–10 cm were taken in the rhizosphere of the potato crops with a geological drill, while the replicates from the same plot were pooled into one composite sample. For comparability, soil cores were only taken from the ridge, as this is the common area of water and fertilizer application for both types of irrigation (sprinkler and drip irrigation). Total genomic DNA was extracted using the FastDNATM Spin Kit for Soil (MP Biomedicals GmbH, Eschwege, Germany) as specified by the manufacturer. For each soil sample per plot, three DNA extractions were carried out. The different microbial-mediated pathways within the N cycle, especially those related to N_2_O emissions, were analyzed by using pathway-specific quantitative real-time polymerase chain reaction (qPCR) approaches to quantify the gene copy numbers per gram of soil of *amo*A (encoding ammonium monooxygenase), nxrB (encoding nitrite oxidoreductase), narG (encoding nitrate reductase), *nir*S/*nir*K (encoding nitrite reductase), and nosZ (encoding N_2_O reductase). The following specific primer sets were used: *amo*A3F/*amo*A-5R for amoA, (amplicon size: 238 bp), nxrB169f/nxrB638r for nrxB (amplicon size: 484 bp), narG572f/narG773r for narG (amplicon size: 201 bp), F1aCU/R3CU for nirK (amplicon size: 472 bp), Cd3aF/R3cd for *nir*S (amplicon size: 416 bp), and nosZ2F/mosZ2R for nosZ amplicon size: 700 bp) (for details, see Supporting Information Table S4 in [28]).

### 2.5. Statistical Analysis

The individual seasonal development of N_2_O fluxes per treatment was analyzed by an ANOVA, based on a generalized linear mixed effect model using the ‘glmmTMB’ [44] package in the R statistical software R version 4.3.3 [45], including the RStudio program [46]. The effects of the different irrigation and fertilization treatments on cumulative area-related N_2_O emissions, crop yields, and yield-related N_2_O emissions were analyzed using ANOVA followed, if necessary, by a Tukey post hoc test using the R [45] and RStudio (version: 2023.12.0+369) [46] statistical software packages. Diagnostic plots were used in order to verify whether the model assumptions were met [47]. For the time-dependent determination of microbial impacts on N_2_O flux rates within the different treatments, detected gene copy numbers were transformed using sqrt transformation before analysis by applying a generalized linear model using the ‘nlme’ packages [48], followed by a two-way ANOVA with a Tukey post hoc test. The distinct sample dates for microbial analysis were unevenly spaced in time. Therefore, an exponential correlation (corEXP) function was fitted to model the temporal correlations between N_2_O emissions and microbial data. For time-independent analysis, Pearson’s correlations were performed by using the ‘rstatix’ package [49] in the R statistical software [45] to explore the relationships between environmental factors, microbial gene copy numbers, and N_2_O emissions. Additionally, a nonmetric multidimensional scaling (NMDS) using the ‘vegan’ package [50] was used to identify the main influencing factors on functional genes. Therefore, data were transformed using square root transformations and standardized using Wisconsin double standardization.

## 3. Results and Discussion

### 3.1. N_2_O Flux Rates Influenced by Different Irrigation and Fertilization Regimes

**Figure 1:**
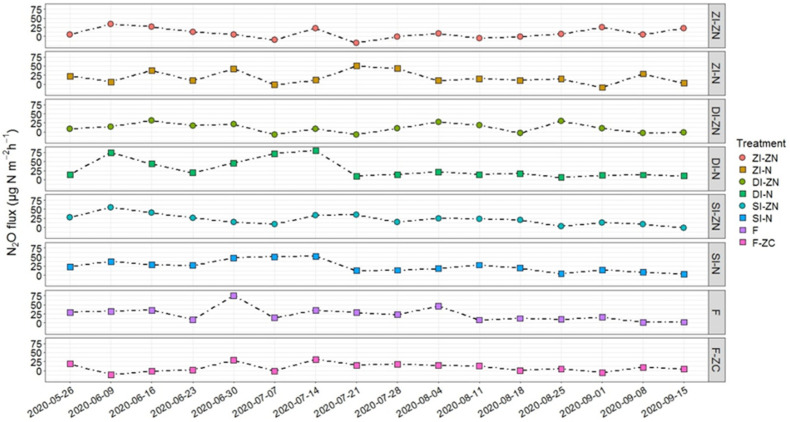
Median N_2_O flux rates during the potato cropping season in 2020 for the different treatments: ZI-ZN = zero irrigation without (zero) nitrogen (N) fertilizer, ZI-N = zero irrigation with broadcasted nitrogen (N) fertilizer, SI-ZN = sprinkler irrigation without (zero) nitrogen (N) fertilizer, SI-N = sprinkler irrigation with broadcasted nitrogen (N) fertilizer, DI-ZN = drip irrigation without (zero) nitrogen (N) fertilizer, DI-N = drip irrigation with broadcasted nitrogen (N) fertilizer, F = fertigation, F-ZC = fertigation without (zero) crops. Information on the application dates and amounts of irrigation water and nitrogen fertilizer is provided in Table S1.

The seasonal median N_2_O fluxes across all treatments ranged from 8.16 μg N_2_O-N m^−2^ h^−1^ (ZI-ZN) to 23.58 μg N_2_O-N m^−2^ h^−1^ (F) (Figure 1, Supplementary Table S3) and were similar to those found on the same study site in 2019 [28]. The statistical analysis did not reveal significant differences, neither for the area-related nor the yield-related cumulative N_2_O emissions, between the investigated treatments (Table 2). The lowest area-related N_2_O emissions originated from the non-irrigated and drip-irrigated plots (ZI-ZN: 0.4 kg N_2_O-N ha^−1^; DI-ZN: 0.38 kg N_2_O-N ha^−1^), while the median cumulative area-related N_2_O emissions on sprinkler-irrigated plots were approximately 65% higher (SI-ZN: 0.63 kg N_2_O-N ha^−1^) (Table 2). The higher N_2_O flux rates detected under sprinkler irrigation (SI-ZN) compared to those of the drip-irrigated plots (DI-ZN) and the untreated reference plots (ZI-ZN) (Figure 1, Supplementary Table S3) confirm the first hypothesis and are in line with a global meta-analysis conducted by Kuang et al. (2021) [3].

Regarding fertilizer application in ZI-N, SI-N, DI-N, and F, an increase in N_2_O flux rates by a factor of two to three compared to the untreated reference treatment (ZI-ZN) was found (Supplementary Table S3). The detected N_2_O fluxes partly exceeded the N_2_O flux rates and cumulative area-related N_2_O emissions found in other studies conducted in potato cropping in sandy soils [36,39,51]. However, comparing these values with those found in 2019 on the same experimental site, lower N_2_O fluxes were detected in this study [28].

Comparing the fertilization application under the different irrigation systems, the highest N_2_O flux rates were detected under sprinkler irrigation (SI-ZN and SI-N), where the highest water volume was applied (Figure 1, Supplementary Tables S1 and S3). It has been shown that potato crop stages are characterized by different nutrient demands, and N is mainly required during later growth stages [3,7,52,53]. Therefore, an adaptation of the rate and timing of fertilization and irrigation based on the actual crop demand could also mitigate N_2_O emissions [10]. Compared to the sprinkler-irrigated plots, N_2_O fluxes were lower in drip-irrigated plots (DI-ZN, DI-N and F), while the application of dissolved N fertilizer directly into the root zone in multiple small applications during crop growth (F) had no further positive effects on N_2_O flux reduction (Figure 1, Supplementary Table S3). This latter observation might be attributed to the different nutrient uptake capacities during the potato growth stages, with appr. 15% during the vegetative stage, 30% during tuber initiation stage, and 58–71% during the tuber bulking stage [52,54]. This indicates that even under fertigation, which is believed to improve N uptake by applying several small fertilizer doses, the supply of N fertilizer could be better adapted to crop demand. However, contrary to the hypothesis, the lowest N_2_O flux rates were detected for the F-ZC treatment (fertigation without (zero) crops), although the N supplied was exclusively available for the microbial community. This indicates the impact of the cultivated crops and their interaction with N_2_O-producing microorganisms. There is evidence that crops affect the assemblage of the microbiome in the soil and rhizosphere by serving as a carbon source for microorganisms, e.g., [55,56]. Moreover, root exudates also affect N transformation processes, with inter alia inhibiting effects on nitrification and denitrification processes [31,53,57,58].

However, in all investigated treatments, except for the untreated reference plots ZI-ZN and the fertigated plots without crops (F-ZC), higher N_2_O flux rates were detected on average during the first half of the cropping season, while N_2_O flux rates decreased in the second half of the growing season (Supplementary Table S3). This is in accordance with results for maize and wheat, where more than 60% of total seasonal N_2_O fluxes occurred during the vegetative stage [59]. Such observations might be inter alia explained by a higher N availability for microorganisms during the vegetative stage, when potato crops have a lower nutrient uptake capacity [52,54]. This assumption is supported by this study, where the percentage N content of the potato tuber increased from June to September in the fertilized treatments compared to the unfertilized ones (Figure S3). Despite this, the N contents of the potato tuber were similar between the respective treatments receiving N fertilizer, and the highest crop yields, with a median value of 48.2 t ha^−1^, were determined for the SI-N treatment (Table 2).

Regarding nitrogen use efficiency (NUE), a range between 42.6 kg_yield_ kg_N_^−1^ for ZI-N and 141.8 kg_yield_ kg_N_^−1^ for SI-N was detected. Comparing the NUE under different irrigation strategies, the lowest NUE values were analyzed for DI-N (81.6 kg_yield_ kg_N_^−1^) and F (68.5 kg_yield_ kg_N_^−1^) compared to the sprinkler-irrigated treatment (SI-N). For the apparent nitrogen recovery (ANR) at harvest, values of 39.5% (ZI-N), 56.8% (SI-N), 42.7% (DI-N) and 33.7% (F) were calculated. All values (crop yields, NUE and ANR) are in accordance with previously published results for potato cropping systems [7,43,52,60], indicating an insufficient use of the applied fertilizer independently of the type of fertilizer application.

### 3.2. Time-Related Effects on Functional Genes Within the Bacterial N Cycle in Applied Management Regimes

**Figure 2:**
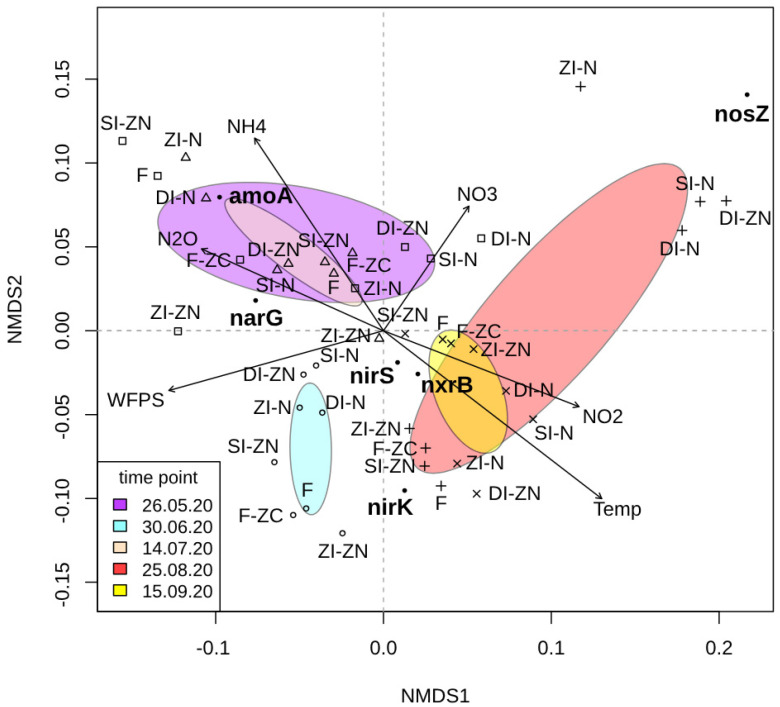
Nonmetric multidimensional scaling (NMDS) analysis based on the detected copy numbers per gram of soil for the investigated genes *amo*A (encoding ammonium monooxygenase), *nxr*B (encoding nitrite oxidoreductase), *nar*G (encoding nitrate reductase), *nir*K/*nir*S (encoding nitrite reductase), and *nos*Z (encoding nitrous oxide reductase). Each color symbolizes one of the investigated treatments:ZI-ZN (no (zero) irrigation without (zero) nitrogen (N) fertilizer), ZI-N (no (zero) irrigation with broadcasted nitrogen (N) fertilizer), SI-ZN (sprinkler irrigation without (zero) nitrogen (N) fertilizer), SI-N (sprinkler irrigation with broadcasted nitrogen (N) fertilizer), DI-ZN (drip irrigation without (zero) nitrogen (N) fertilizer), DI-N (drip irrigation with broadcasted nitrogen (N) fertilizer), F (fertigation), lF-ZC (fertigation without (zero) crops). Symbols are used to indicate the different time points (square = 26 May 2020, diamond = 30 June 2020, triangle = 14 July 2020, plus = 25 August 2020, cross = 15 September 2020). The ellipses represent the time points and their respective treatment variants belonging to the different time points, with a 95% certainty (purple = 26 May 2022, light blue = 30 June 2020, bisquel = 14 July 2020, red = 25 August 2020, yellow = 15 September 2020). The investigated functional genes are marked with a black bold dot (●). The given environmental vectors (arrows) symbolize the measured environmental parameters in terms of soil temperature (Temp), water filled pore space (WFPS), soil ammonium content (NH_4_^+^), soil nitrite content (NO_2_^−^), and the N_2_O flux rates (N_2_O).

A nonmetric multidimensional scaling (NMDS) was carried out to identify (dis-)similarities between the different management systems over the entire growing season based on the quantities of the six investigated functional genes per sampling point (Figure 2). Additionally, the inserted arrows indicate the influence of the investigated environmental variables and their effects on the functional genes, separated by time point and management system. Independent of the investigated treatment effects, this NMDS analysis revealed a time-dependent clustering, separating the first and the second half of the season. Regarding the first half of the season, it was shown that the quantities of detected *amo*A and *nar*G gene copy numbers per gram of soil were mostly responsible for the ordination configuration of the samples. Additionally, this ordination configuration could be correlated with the measured N_2_O fluxes. This leads to the assumption that the detected N_2_O flux rates were pre-dominant during the first half of the season, as N_2_O, as the dependent variable, could be explained by the respective ordination scores (Figure 2). As the gene quantities of *amo*A and *nar*G were also correlated within this ordination configuration, it might be feasible that the measured N_2_O fluxes during the first half of the season were probably a product of a coupled nitrification–denitrification process. This observation is supported by several studies showing that fertilizer application favors ammonia-oxidizing bacteria and, correspondingly, might enhance the potential of nitrification-derived N_2_O production [4,61,62,63]. Moreover, it has been shown that potato crops have different nutritional demands at different growth stages, with lower uptake capacity during the early vegetative stage [31,52,54,64]. Due to the lower nutrient uptake, and hence higher N availability for microorganisms, the higher flux rates in the first half of the season could be explained.

### 3.3. Effects of Different Irrigation Technologies on the Bacterial N Cycle

**Figure 3:**
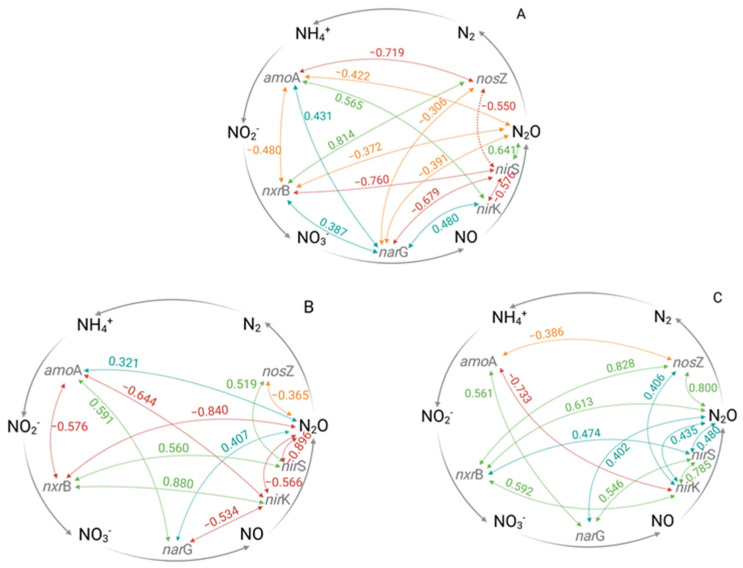
Pearson’s correlations for the detection of time-independent relationships between the recorded gene copy numbers per gram of soil and N_2_O flux rates of the ridge (0–10cm). Weak (≥0.30; positive: blue, negative: orange), moderate (≥0.50, positive green, negative: red) and strong (≥0.75, positive: green, negative: red) correlations are shown. (**A**) ZI-ZN (zero irrigation without (zero) nitrogen (N) fertilizer), (**B**) SI-ZN (sprinkler irrigation without (zero) nitrogen (N) fertilizer), (**C**) DI-ZN (drip irrigation without (zero) nitrogen (N) fertilizer, *amo*A = gene encoding ammonium monooxygenase, *nxr*B = gene encoding nitrite oxidoreductase, *nar*G = gene encoding nitrate reductase, *nir*K/*nir*S = genes encoding nitrite reductase and *nos*Z = gene encoding nitrous oxide reductase.

In general, the microbial community composition is influenced by several environmental factors that fluctuate seasonally and temporally, causing highly variable N_2_O fluxes, e.g., [13,14,24,28]. One of the most important driving factors for N_2_O release from soils is the availability of oxygen (O_2_), which negatively correlates with soil water content [13,14,65]. In this regard, irrigation in general leads to a temporal and spatial increase in the soil water content, and hence anaerobic conditions, which might stimulate microbial N_2_O production via the denitrification pathway [3,13,14,66], although it has to be considered that these effects can be short-term. For the genetically determined denitrification process, the three enzymes, nitrate reductase, nitrite reductases, and nitrous oxide reductase, with their corresponding genes *nar*G, *nir*K/*nir*S, and *nos*Z, are frequently considered in relation to N_2_O production [14,24]. Regarding the seasonal development of the functional gene quantities in this study, in the untreated reference plots (ZI-ZN), a significant decrease in the *nar*G gene copy number g^−1^ soil over the cropping season was detected, while no significant seasonal changes were found in either the *nir*K or the *nir*S gene copy number g^−1^ soil. The *nos*Z gene copy number g^−1^ soil significantly decreased during the second half of the season (Supplementary Figure S3 and Table S5). Additionally, Pearson analysis revealed a moderate negative correlation between the *nir*S and *nos*Z gene copy numbers g^−1^ soil (r = −0.550), but a strong positive correlation between the *nir*S gene copy number g^−1^ soil and the detected N_2_O flux rates (r = 0.641) (Figure 3A, Supplementary Table S6). Therefore, the microbial community in the untreated reference ZI-ZN seemed to be dominated by *nir*S-type microorganisms, which potentially were responsible for the detected N_2_O fluxes. However, as most of the detected correlations between the investigated genes were negative, a clear pathway of the N_2_O formation cannot be derived (Figure 3A, Supplementary Table S6).

The highest N_2_O flux rates were detected under sprinkler irrigation (SI-ZN) conditions, suggesting that the increased soil water content stimulated N_2_O production, particularly during the first half of the season (Figure 1). Contrary to the hypothesis that higher water volumes stimulate the process of denitrification, the detected correlation patterns of the investigated genes showed that denitrification was unlikely to be the potential underlying pathway of N_2_O production under SI-ZN (Figure 3B, Supplementary Table S7). This might be explained by the low incidence of water-filled pore spaces (WFPS), which did not exceed 28% (Figure S2), and hence did not reach the optimal conditions for denitrification (70–80% WFPS) [13,14,27,67,68]. The low WFPS values can be attributed to the low water-holding capacity of sandy soils [3]. Therefore, it is more probable that soils with fluctuating aerobic-anaerobic conditions under sprinkler irrigation systems promote N_2_O production by nitrifier denitrification [25]. During the first step, NH_4_^+^ is oxidized to hydroxylamine (NH_2_OH), which is further oxidized to nitric oxide (NO) [14,24,26,69] and subsequently to N_2_O [25,26]. In this regard, the *amo*A gene copy number g^−1^ soil showed a significant increase during the first half of the cropping season (Supplementary Figure S4 and Table S5), while the Pearson analysis revealed a positive correlation with the detected N_2_O flux rates (r = 0.321) (Figure 3B, Supplementary Table S7). In addition, the *amo*A gene copy number g^−1^ soil was negatively correlated with the *nxr*B gene copy number g^−1^ soil (r = 0.576) but positively correlated with the *nar*G gene copy number g^−1^ soil (r = 0.591) (Figure 3B, Supplementary Table S7). It has to be noted that the nxr and nar genes show structural similarities, and hence their corresponding enzymes most likely have comparable metabolic capacities, as they not only convert NO_2_^−^ to NO_3_^−^ but they might also enable the oxidation of NO_3_^−^ to NO_2_^−^ [22,70]. However, the *nxr*B gene copy number g^−1^ soil positively correlated with *nir*K and the *nir*S gene copy numbers g^−1^ soil (r = 0.880, r = 0.560), while the *nir*S gene copy numbers g^−1^ soil, in turn, positively correlated with the *nos*Z gene copy number g^−1^ soil (r = 0.519) (Figure 3B, Supplementary Table S6). Moreover, the *nir*K and the *nir*S gene copy numbers g^−1^ soil further had a moderate to strong correlation with the detected N_2_O flux rates (r = −0.566, r = −0.896) (Figure 3B, Supplementary Table S7). Therefore, it can be assumed that the N_2_O release of SI-ZN might not be related to the process of denitrification but is more likely related to the process of nitrifier denitrification. Apart from microbial-mediated N_2_O production, chemical reactions could also contribute to total N_2_O emissions, especially under acidic soil conditions (pH < 4.5) [14,64,71]. However, the chemical process can almost be ruled out for our study site, which is characterized by a pH value of 6.75.

In contrast to SI-ZN and in accordance with the first hypothesis, the process of denitrification under drip irrigation (DI-ZN) seems to be feasible, as the Pearson analysis revealed positive correlations either between the *nxr*B and the *nir*K gene copy numbers g^−1^ soil (r = 0.592) or the *nar*G and the *nir*S gene copy numbers g^−1^ soil (r = 0.546). The gene copy numbers g^−1^ soil of *nxr*B and *nir*K were at the same level throughout the entire season and could further be correlated with the N_2_O flux rates (r = 0.613, r = 0.435) (Figure 3C, Supplementary Table S8). This indicates that the occurring microbial community was most probably dominated by *nir*K-type microorganisms, which often lack the *nos*Z gene, and hence produce N_2_O as their denitrification end product [68,72]. In that case, it can be assumed that higher soil moisture near the drippers leads to temporal and special anaerobic conditions, which might favor the process of denitrification, whereas the higher frequency of wet–dry cycles around the drippers might further enhance N_2_O production. Therefore, it can be concluded that water application might stimulate N_2_O production compared to the control treatment (ZI-ZN). However, an increase in N_2_O flux rates due to water application cannot exclusively be related to the process of denitrification based on the detected quantities of the investigated functional gene of the N cycle. The Pearson analysis also indicated a potential contribution of the process of nitrifier denitrification, which stands partly in contrast with the first hypothesis.

### 3.4. Effects of Different N Fertilizer Application Technologies on the Bacterial N Cycle

N fertilizer addition (ZI-N, SI-N, DI-N and F) increased N_2_O flux rates by a factor of two to three compared to the untreated reference ZI-ZN, whereas the application type of water and fertilizer (SI-N, DI-N and F) had only minor effects on the N_2_O flux rates (Figure 1, Supplementary Tables S3 and S4).

**Figure 4:**
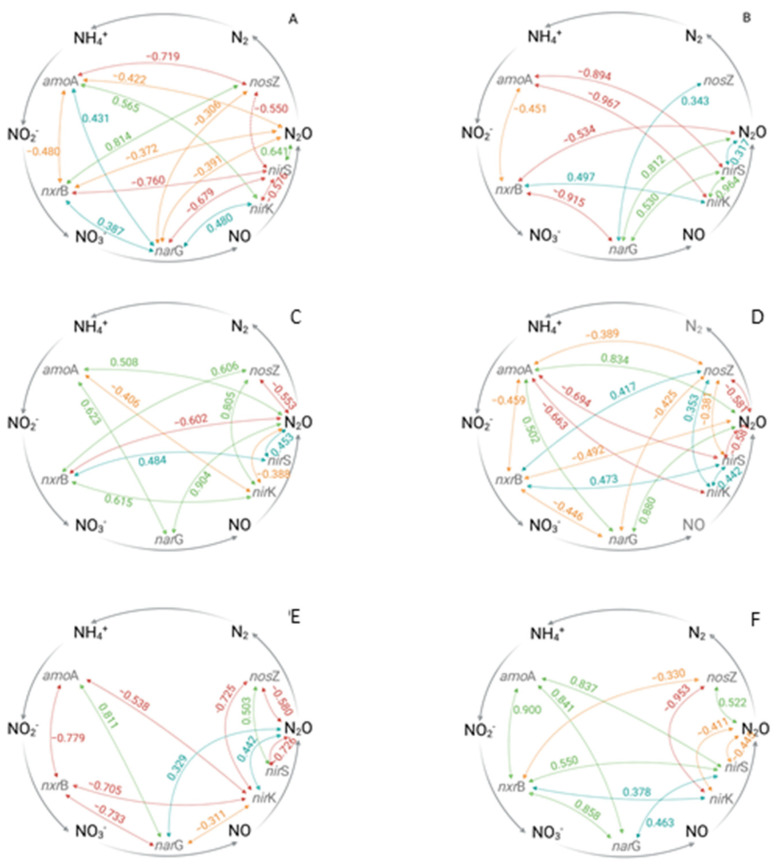
Pearson’s correlations for the detection of time-independent relationships between the recorded gene copy numbers per gram of soil and N_2_O flux rates of the ridge (0–10cm). Weak (≥0.30; positive blue, negative: organge), moderate (≥0.50, positive green, negative: red) and strong (≥0.75, positive green, negative: red) relationships are shown. (**A**) ZI-ZN (zero irrigation without (zero) nitrogen (N) fertilizer), (**B**) ZI-N (zero irrigation with broadcasted nitrogen (N) fertilizer), (**C**) SI-N (sprinkler irrigation with broadcasted nitrogen (N) fertilizer), (**D**) DI-N (drip irrigation with broadcasted nitrogen (N) fertilizer), (**E**) F (fertigation), (**F**) F-ZC (fertigation without (zero) crops), amoA = gene encoding ammonium monooxygenase, nxrB = gene encoding nitrite oxidoreductase, narG = gene encoding nitrate reductase, nirK/nirS = genes encoding nitrite reductase and nosZ = gene encoding nitrous oxide reductase.

A clear effect of the N fertilizer addition, irrespective of additional water supply, was found in non-irrigated and broadcasted fertilized treatments (ZI-N), where N_2_O flux rates were two-fold higher compared to the untreated reference ZI-ZN (Supplementary Tables S3 and S4). While no clear potential pathway for N_2_O formation could be derived for the untreated reference ZI-ZN, the correlation pattern under broadcasted N application suggests that the N_2_O production under ZI-N could have been based on the denitrification process. A positive correlation was either found between the nxrB and nirK gene copy numbers g^−1^ soil (r = 0.497) or between the narG and nirS gene copy numbers g^−1^ soil (r = 0.530) (Figure 4B, Supplementary Table S9). Additionally, the Pearson analysis revealed a strong positive correlation between the denitrification genes nirK and nirS (r = 0.964), while the *nir*S gene itself further positively correlated to the detected N_2_O flux rate (r = 0.317) (Figure 4B, Supplementary Table S9). ANOVA analysis further revealed a significant relationship between both the *nar*G and the *nir*S genes gene copy numbers g^−1^ soil and the N_2_O flux rates (*p* = 0.008, *p* = 0.085) for ZI-N (Supplementary Table S14). The detected quantities of both genes showed a significant increase during the first half of the season followed by a significant decrease. These findings are in accordance with results of a meta-analysis, which identified a stimulating effect of N amendment inter alia on the quantities of bacterial denitrification genes [73].

Moreover, water supply, either by sprinkler irrigation (SI-N) or drip irrigation (DI-N, F), only led to a minor further increase in the N_2_O flux rates (Figure 1, Supplementary Tables S3 and S4). Regarding the effects of broadcasted N applications under different irrigation systems (SI-N, DI-N) on the genetically determined functional gene composition, differences in the correlation pattern of the investigated genes, and hence the possible microbial N_2_O production pathways, were found (Figure 4C,D). Surprisingly, the broadcasted N fertilizer application under sprinkler irrigation (SI-N) led to a slight reduction in the N_2_O flux rates (Figure 1, Supplementary Tables S2 and S3). Based on the positive correlation between the amoA and *nar*G gene copy numbers g^−1^ soil (r = 0.623), as well as their individual correlation with the detected N_2_O flux rates (r = 0.508, r = 0.904), a nitrifier denitrification can be assumed (Figure 4C, Supplementary Table S10). Additionally, positive correlations were found between the *nxr*B gene copy number g^−1^ soil and both denitrification genes *nir*K and *nir*S (r = 0.615, r = 0.484), with a subsequent positive correlation between the *nir*S gene copy number g^−1^ soil and the N_2_O flux rates (r = 0.453) (Figure 4C, Supplementary Table S9). Therefore, greater N_2_O flux rates might have been expected under SI-N compared to SI-ZN. However, the strong positive correlation between the *nir*K and the *nos*Z gene copy numbers g^−1^ soil (r = 0.805) (Figure 4C, Supplementary Table S10) indicates a transformation of N_2_O into N_2_, resulting in lower N_2_O flux rates under SI-N. This is in accordance with the study by You et al. (2022), who detected inter alia an increase in *nir*K and *nos*Z under N amendment [73].

In contrast to the sprinkler-irrigated treatments (SI-ZN and SI-N), the broadcasted as well as the dissolved N fertilizer supply under drip irrigation (DI-N and F) resulted in increased N_2_O formation (Figure 1, Supplementary Tables S3 and S4), which stands in contrast to the second hypothesis and the results provided by Kuang et al. (2021) [3]. However, a greater frequency of wetting and drying cycles next to the dripper could enhance N_2_O production. Therefore, the higher N_2_O flux rates under drip irrigation in this study could be explained. N_2_O release under drip-irrigated unfertilized treatment (DI-ZN) was most probably attributed to a *nxr*B/*nir*K-type dominated bacterial community, whereas under DI-N and F, no clear N_2_O production pathway based on the investigated gene quantities and their correlation pattern could be found. Both treatments showed a positive correlation between the *amo*A and *nar*G gene copy numbers g^−1^ soil (r = 0.502 for DI-N, r = 0.811 for F), with subsequent a correlation between the *nar*G gene copy number g^−1^ soil and the detected N_2_O flux rates (r = 0.880 for DI-N, r = 0.329 for F) (Figure 4D,E, Supplementary Tables S11 and S12). This indicates that N_2_O production might be related to the process of nitrifier denitrification, which is in accordance with the enhancing effects on nitrifier denitrification due to changes between aerobic and anaerobic soil conditions [25] as they occur next to the drippers.

The lowest N_2_O flux rates were measured for the F-ZC treatment (fertigation without (zero) crops), which is most probably related to the absence of potato crops, and hence the non-availability of carbon sources (root exudates) for the soil microorganisms, e.g., [31,53,55,58,59,60]. However, the correlation pattern of the investigated genes indicated that both the process of nitrification and denitrification could have been carried out (Figure 4E). For example, the Pearson analysis revealed positive relationships between the amoA gene copy number g^−1^ soil and the *nxr*B and *nar*G gene copy numbers g^−1^ soil (r = 0.900, r = 0.841), which are indicative of the nitrification pathway, whereas the positive correlations between the *nxr*B and both *nir*K and nirS gene copy numbers g^−1^ soil (r = 0.378, r = 0.550), as well as between the *nar*G and *nir*S gene copy numbers g^−1^ soil (r = 0.463), indicated the potential for the denitrification pathway (Figure 4E, Supplementary Table S13). However, the negative correlations of the *nir*K and *nir*S gene copy numbers g^−1^ soil and the N_2_O flux rates might explain the low N_2_O flux rates in general (r = −0.411, r = −0.445) (Figure 4E, Supplementary Table S13).

### 3.5. Summarized Assessment of the Genetically Determined N_2_O Production Pathways Within Applied Management Regimes

This study elucidated the potential occurrence of bacterial-mediated pathways of N_2_O release from sandy agricultural soils affected by different irrigation and nitrogen fertilization regimes in potato cropping. The N_2_O flux rates in this study were mostly affected by the amount of additionally supplied water, with the highest N_2_O flux rates being found under sprinkler irrigation (received 120 L m^−2^) conditions compared to drip irrigation (received 37 L m^−2^) and fertigation (received 57 L m^−2^), instead of the type and mode of N fertilizer application (broadcasted application vs. dissolved in irrigation water; all received 150 kg N ha^−1^). These differences in the detected N_2_O flux rates are generally in accordance with the first hypothesis that an additional water supply would lead to higher N_2_O fluxes. However, the correlation patterns of the quantities of the investigated functional genes and their correlation with N_2_O flux rates revealed that higher water volume application did not result in stimulating the denitrification processes but rather the process of nitrifier denitrification.

A comparison of the effects of only sprinkler irrigation (SI-ZN) and only N fertilizer (ZI-N) on the potential pathways of N_2_O formation revealed two different options in terms of nitrifier denitrification for SI-ZN or an *nir*S-type denitrification for ZI-N, whereas the combined application of sprinkler irrigation and N fertilizer (SI-N) potentially promoted N_2_O production related to both pathways. Moreover, the comparison of the different water application types (sprinkler vs. drip irrigation; SI-ZN vs. DI-ZN) indicated a predominant *nxr*B-*nir*K-type denitrification under drip irrigation, which is most probably related to more pronounced anaerobic conditions due to higher soil moisture near the drippers. Therefore, the first hypothesis can only partly be proven in this study. The type of N fertilizer supply—broadcasted application or dissolved in irrigation water (DI-N vs. F)—showed only minor differences in potential microbial community functionality. N_2_O production in both treatments was most probably dominated by nitrifier denitrification, while for DI-N, a *nxr*B-*nir*K/*nir*S-type denitrification might also be feasible. This stands in contrast to the hypothesized better N use efficiency under fertigated systems resulting in lower N_2_O emissions.

However, generally higher N_2_O flux rates were detected during the first half of the cropping season due to higher N availability for microorganisms during the vegetative stage of the potatoes, while the N_2_O flux rates decreased during the second half of the season, when the potato crops had a higher N uptake. In this regard, similarity analyses revealed that the potential bacterial-mediated N_2_O release might have been dominated by nitrifier denitrification during the first half of the season due to the fact that fertilizer application favors ammonia-oxidizing bacteria and correspondingly enhances the potential of nitrification-derived N_2_O production.

## 4. Conclusions

The applied irrigation and fertilization technologies in this study led to different N_2_O flux rates over the entire cropping season, with higher flux rates during the first half of the season accompanied by a low NUE and ANR of the provided N fertilizer by the potato crops. Regarding the potentially underlying genetically determined N_2_O production pathway, this study indicates that the nitrifier denitrification process might be of great importance. Further research is required to adjust the amount and time of water and N fertilizer application based on crop demand for nutrients, and their related nitrogen use efficiency and apparent nitrogen recovery during the different crop growth stages, in order to mitigate N_2_O release from agriculture. This could be achieved by a constant monitoring of the N_2_O flux rates, using automated gas measurements with a higher temporal resolution to determine N_2_O emissions more accurately. Moreover, research is needed that combines the quantification of functional genes of the N cycle with the occurring taxonomic profiles of the microbial communities, as well as with the occurring N_2_O pathways determined by isotopic approaches. Considering this, management measures of cropping systems can be improved with regard to the nutrient use efficiency and apparent nutrient recovery rates of cultivated crops through the targeted control of the occurring microbial-mediated regulation mechanisms in nutrient cycles, and hence mitigate N_2_O emissions from agricultural systems.

## Figures and Tables

**Table 1:** Variants of irrigation and fertilization with corresponding temporal and spatial distributions of water and nitrogen supply. For details, see Table S1 (Supplementary Material), which gives an overview of the water and nitrogen (N) fertilizer applications with indications of the date and amount during the season.

Variant	Irrigation	N Fertilization	Distribution of Water Supply		Distribution of N Supply	
			Temporal	Spatial	Temporal	Spatial
ZI-ZN	no irrigation	no N fertilization	stochastic (rainfall)	homogeneous	stochastic (mineralization of organic matter)	homogeneous
ZI-N	no irrigation	N fertilization (optimal 150 kg/ha)	stochastic (rainfall)	homogeneous	three times per season (75 kg/ha first-order foliation, 45 kg/ha beginning of tuberization and 30 kg/ha maturation)	homogeneous
SI-ZN	sprinkling irrigation	no N fertilization	irregularly in addition to rainfall	homogeneous	stochastic (mineralization of organic matter)	homogeneous
SI-N	sprinkling irrigation	N fertilization (optimal 150 kg/ha)	irregularly in addition to rainfall	homogeneous	three times per season (75 kg/ha first-order foliation, 45 kg/ha beginning of tuberization and 30 kg/ha maturation)	homogeneous
DI-ZN	drip irrigation	no N fertilization	irregularly in addition to rainfall	punctual in grid knot pattern	stochastic (mineralization of organic matter)	homogeneous
DI-N	drip irrigation	N fertilization (optimal 150 kg/ha)	irregularly in addition to rainfall	punctual in grid knot pattern	three times per season (75 kg/ha first-order foliation, 45 kg/ha beginning of tuberization and 30 kg/ha maturation)	homogeneous
F	fertigation (optimal N fertilization 150 kg/ha)		regularly in short intervals (5 to 15 mm every week)	punctual in grid knot pattern	regularly in short intervals (about 5 to 15 kg/ha every week)	punctual in grid knot pattern
F-ZC	fertigation, no crops (optimal N fertilization 150 kg/ha)		regularly in short intervals (5 to 15 mm every week)	punctual in grid knot pattern	regularly in short intervals (about 5 to 15 kg/ha every week)	punctual in grid knot pattern

**Table 2:** (a) Median values of cumulative area-related N_2_O emissions, yield, and yield-related N_2_O emissions per treatment calculated for the cropping season in 2020. Significant differences between treatments are indicated by different lowercase letters in column Group (‘a’, ‘b’, ‘c’, and ‘d’ ANOVA followed a by Tukey post hoc-test). (b) Modell parameter (F and *p* values) of the ANOVAs for linear models with ‘Cumulative area-related N_2_O emissions’, ‘Yield and Yield-related N_2_O emissions’ as response variables and ‘Treatment’ and ‘Block’ (blocks from a randomized block design) as explanatory variables. Values with *, *** indicate significance at *p* < 0.05, 0.01, 0.001.

		Cumulative Area-Related N_2_O Emissions (kg N_2_O-N ha^−1^)		Yield (t ha^−1^)		Yield-Related N_2_O Emissions (kg N_2_O-N t^−1^ Yield)	
		Median	Group	Median	Group	Median	Group
(a)	ZI-ZN	0.40	a	20.4	a	0.0194	a
	ZI-N	0.68	a	29.4	bc	0.0211	a
	SI-ZN	0.63	a	25.6	ab	0.0248	a
	SI-N	0.61	a	48.2	d	0.0126	a
	DI-ZN	0.38	a	26.8	ab	0.0170	a
	DI-N	0.83	a	30.6	c	0.0213	a
	F	0.60	a	33.7	c	0.0227	a
	F-ZC	0.30	a	-	-	-	-
(b)	ANOVA	F:	*p*:	F:	*p*:	F:	*p*:
	Treatment	2.109	0.111	14.146	<0.001 ***	0.736	0.631
	Block	1.234	0.321	13.954	<0.001 ***	5.532	0.02 *

## Data Availability

The raw data supporting the conclusions of this article will be made available by the authors on request.

## Supplementary Materials

The following supporting information can be downloaded at: https://www.mdpi.com/article/10.3390/microorganisms13040741/s1, Figure S1: Mean daily air temperature and precipitation recorded in season 2020. Figure S2: easonal development of water filled pore spaces. Figure S3: Dry weight and nitrogen content of potato tuber. Figure S4: Determined quantities of log transformed gene copy numbers per gram of soil of *amo*A, *nar*G, *nir*K, *nir*S, *nos*Z. Table S1: Overview of water and nitrogen (N) fertilizer application dates. Table S2: Overview of different nitrogen (N) forms extracted from the Nmin sampling per sampling date for the investigated treatments. Table S3: Seasonal N2O flux rates as median value of three field replicates. Table S4: Modell parameter of linear mixed effects models (a) and Seasonal development of N2O fluxes (b). Table S5: Median values of the gene copy numbers per gram of soil of the nitrogen cycle for the investigated treatments ZI-ZN. Table S6: Pearson’s correlations for the detection of time-independent relationships among the investigated gene copy numbers per gram of soil and between the investigated genes and the main environmental factors for treatment ZI-ZN. Table S7: Pearson’s correlations for the detection of time-independent relationships among the investigated gene copy numbers per gram of soil and between the investigated genes and the main environmental factors for treatment SI-ZN. Table S8: Pearson’s correlations for the detection of time-independent relationships among the investigated gene copy numbers per gram of soil and between the investigated genes and the main environmental factors for treatment DI-ZN. Table S9: Pearson’s correlations for the detection of time-independent relationships among the investigated gene copy numbers per gram of soil and between the investigated genes and the main environmental factors for treatment ZI-N. Table S10: Pearson’s correlations for the detection of time-independent relationships among the investigated gene copy numbers per gram of soil and between the investigated genes and the main environmental factors for treatment SI-N. Table S11: Pearson’s correlations for the detection of time-independent relationships among the investigated gene copy numbers per gram of soil and between the investigated genes and the main environmental factors for treatment DI-N. Table S12: Pearson’s correlations for the detection of time-independent relationships among the investigated gene copy numbers per gram of soil and between the investigated genes and the main environmental factors for treatment F. Table S13: Pearson’s correlations for the detection of time-independent relationships among the investigated gene copy numbers per gram of soil and between the investigated genes and the main environmental factors for treatment F-ZC. Table S14: Time dependent mixed effect model with ‘N2O fluxes’ as response variable and ‘week’ and ‘gene copy numbers per gram soil’ and their interactions as variables.
