# Supplementary material for: Irrigation System, Rather than Nitrogen Fertilizer Application, Affects the Quantities of Functional Genes Related to N2O Production in Potato Cropping
Source: Microorganisms. 2025 Mar 25;13(4):741. doi: 10.3390/microorganisms13040741 (PMC12029621; doi:10.3390/microorganisms13040741)
Supplement: Supplementary file 1 [file microorganisms-13-00741-s001.zip › microorganisms-3477143-supplementary.pdf]

Supplementary materials

Manuscript title: The volume of irrigation water rather than nitrogen fertilizer application affected the quantities of functional genes related to N_2O production in potato cropping

Authors: Laura Charlotte Storch, Katharina Schulz, Jana Marie Kraft, Annette Prochnow, Liliane Rueß, Benjamin Trost, Susanne Theuerl

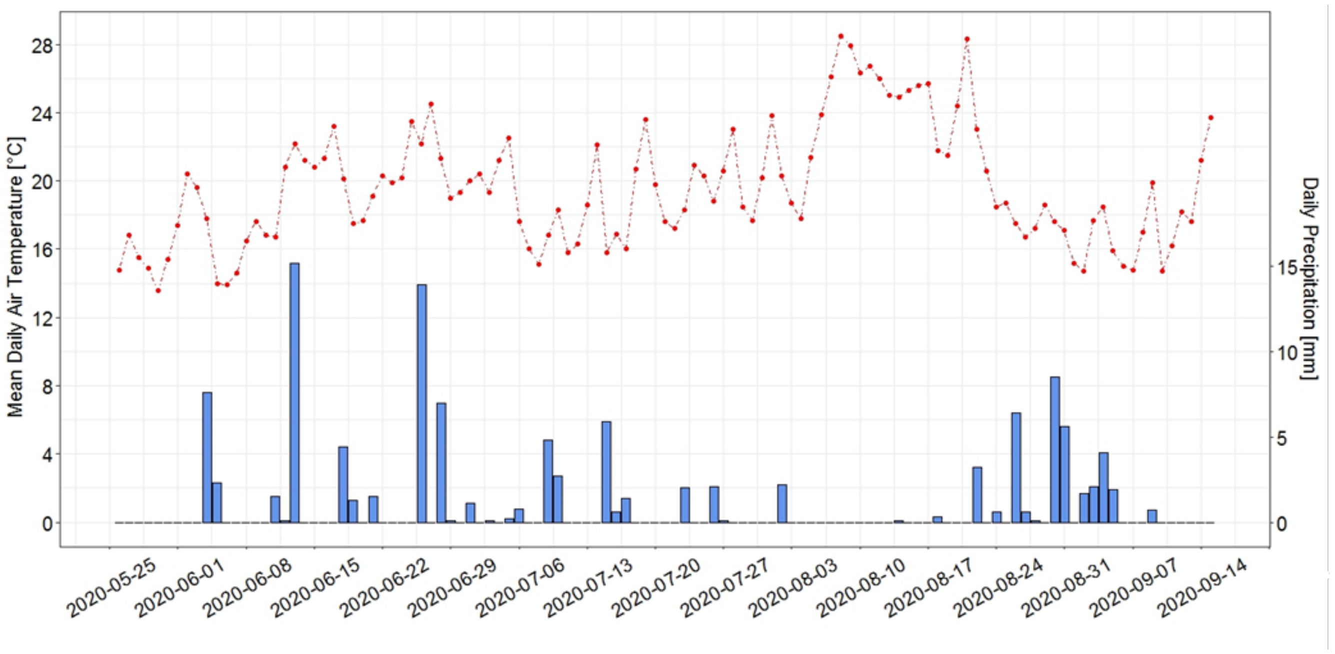

Figure S1: Mean daily air temperature and precipitation recorded between 25th May and 14th September 2020 at the Field Research Station Marquardt of the Leibniz Institute for Agricultural Engineering and Bioeconomy, Brandenburg, Germany.

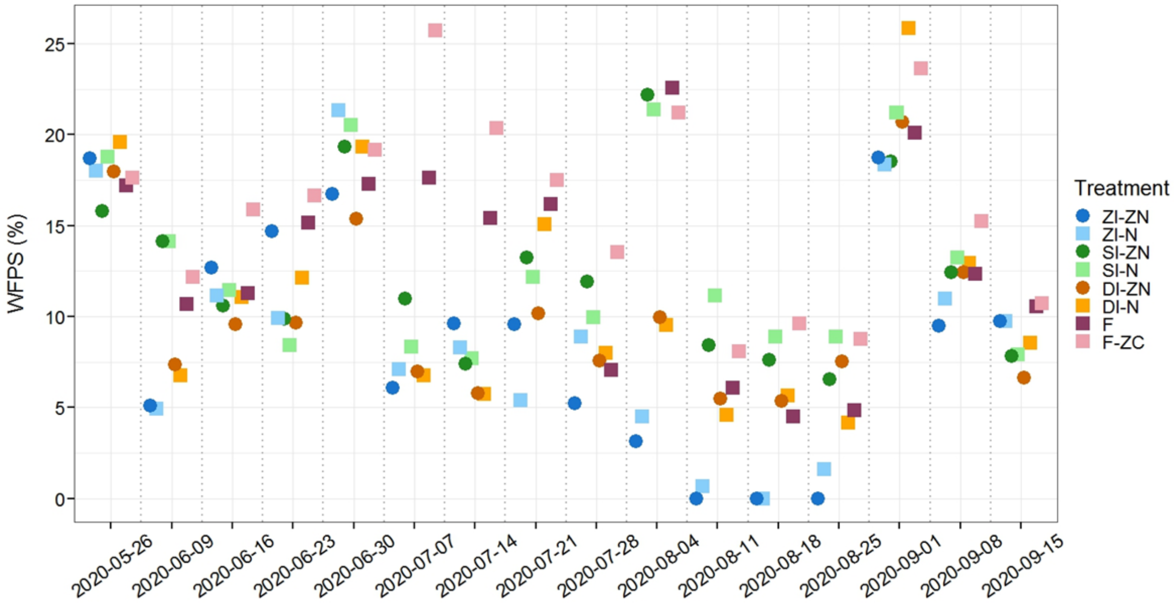

Figure S2: Seasonal development of water filled pore spaces (WFPS; shown are median values) for each treatment. Symbol code: circles = not fertilized treatments, squares = nitrogen fertilized treatments; Color code: dark blue = ZI-ZN (no (zero) irrigation without (zero) nitrogen (N) fertilizer), light blue = ZI-N (no (zero) irrigation with broadcasted N fertilizer), dark green = SI-ZN (sprinkler irrigation without (zero) N fertilizer), light green = SI-N (sprinkler irrigation with broadcasted N fertilizer), dark orange = DI-ZN (drip irrigation without (zero) N fertilizer), light orange = DI-N (drip irrigation with broadcasted N fertilizer), dark purple = F (fertigation), light purple = F-ZC (fertigation without (zero) crops).

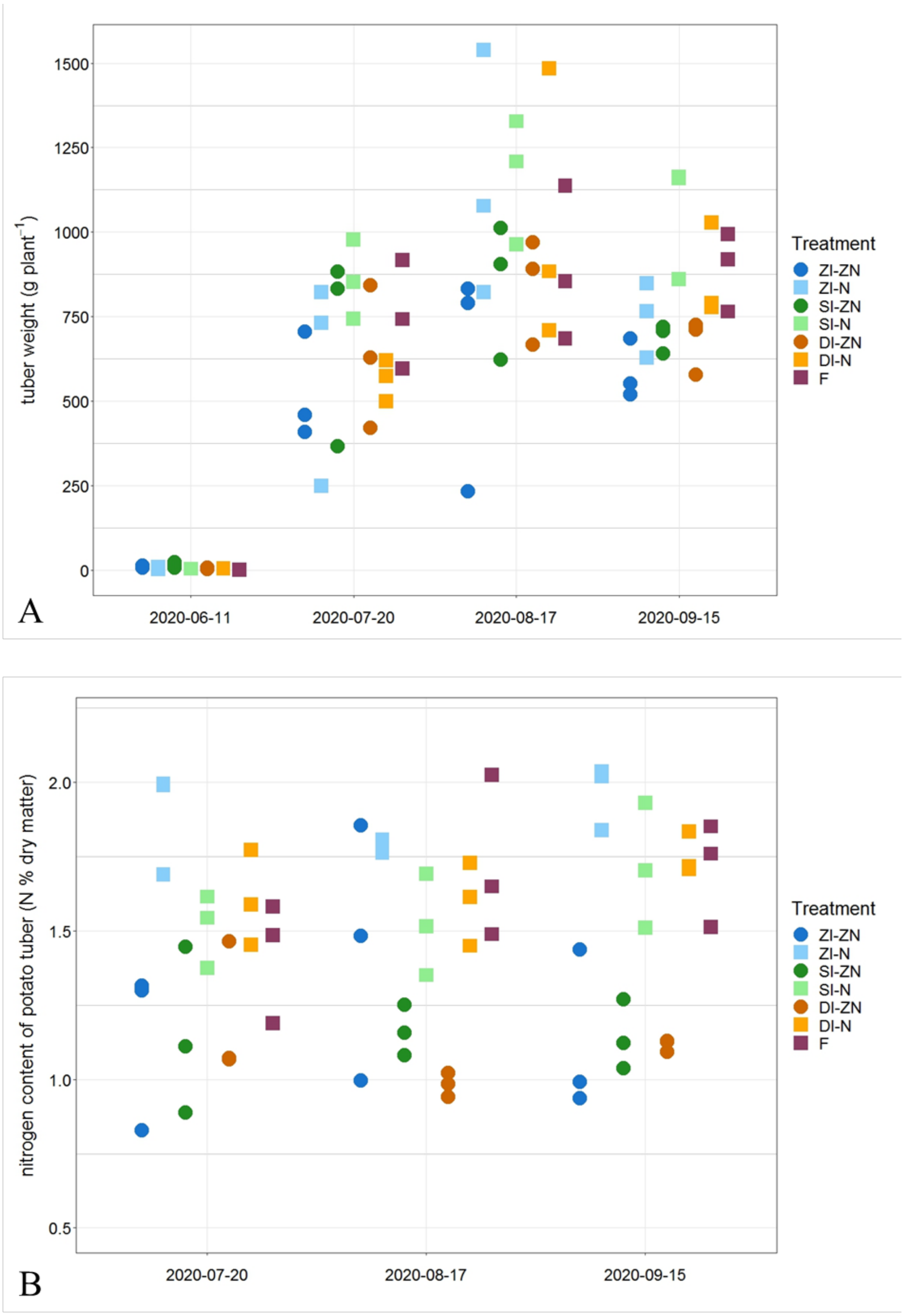

Figure S3: Dry weight [g per plant] and nitrogen content [% dry matter) of potato tuber at four different sampling points (June, July, August and September) during the cropping season 2020. Symbol code: circles = not fertilized treatments, squares = nitrogen fertilized treatments; Color code: dark blue = ZI-ZN (no (zero) irrigation without (zero) nitrogen (N) fertilizer), light blue = ZI-N (no (zero) irrigation with broadcasted N fertilizer), dark green = SI-ZN (sprinkler irrigation without (zero) N fertilizer), light green = SI-N (sprinkler irrigation with broadcasted N fertilizer), dark orange = DI-ZN (drip irrigation without (zero) N fertilizer), light orange = DI-N (drip irrigation with broadcasted N fertilizer), dark purple = F (fertigation), light purple = F-ZC (fertigation without (zero) crops).

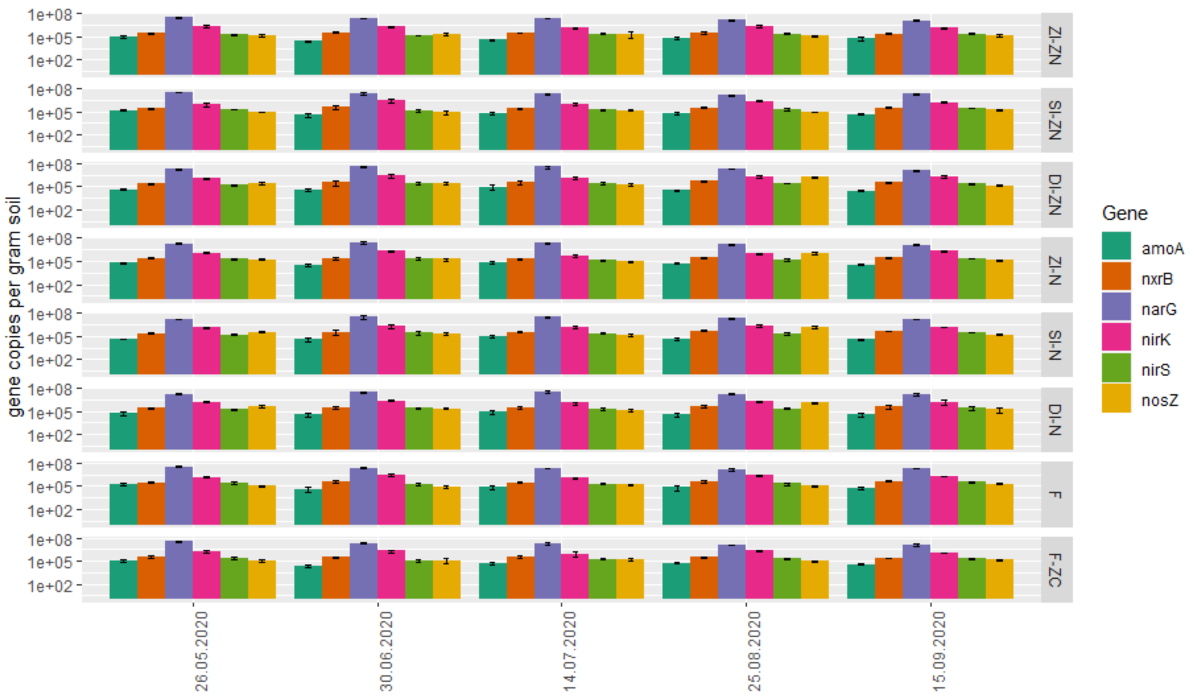

Figure S4: Determined quantities of log transformed gene copy numbers per gram soil with standard deviation of *amo*A (gene encoding ammonium monooxygenase, green), *nxr*B (gene encoding nitrite oxidoreductase, orange), *nar*G (gene encoding nitrate reductase, purple), *nir*K (gene encoding nitrite reductase, pink), *nir*S (gene encoding nitrite reductase, light green), *nos*Z (gene encoding N_2O reductase, yellow) for each treatment: ZI-ZN (no (zero) irrigation without (zero) nitrogen (N) fertilizer), ZI-N (no (zero) irrigation with broadcasted N fertilizer), SI-ZN (sprinkler irrigation without (zero) N fertilizer), SI-N (sprinkler irrigation with broadcasted N fertilizer), DI-ZN (drip irrigation without (zero) N fertilizer), DI-N (drip irrigation with broadcasted N fertilizer), F (fertigation), F-ZC (fertigation without (zero) crops), over the cropping season 2020 (n = 162 per treatment and gene).

Table S1: Overview of water and nitrogen (N) fertilizer application with indication of the date and amount for the investigated treatments ZI-ZN (zero irrigation without (zero) N application), ZI-N (zero irrigation with N application), SI-ZN (sprinkler irrigation without (zero) N application), SI-N (sprinkler irrigation with N application), DI-ZN (drip irrigation without (zero) N application), DI-N (drip irrigation with N application), F (fertigation) and F-ZC (fertigation without (zero) crops). Marked in bold are sampling dates for functional genes analysis.

Sampling date		**26.05.20**	09.06.20	16.06.20	23.06.20	**30.06.20**	07.07.20	**14.07.20**	21.07.20	28.07.20			04.08.20		11.08.20
Air temperature [°C]		19.0	21.1	25.8	24.7	21.2	17.9	28.2	22.0	26,7			24.3		31.0
Soil temperature [°C]		12.6	17.3	19.0	17.9	16.2	15.4	17.3	17.5	19,7			17.1		23.1
Precipitation [mm]		16.3	0.1	12.5	2.3	11.2	3.0	0.0	0.1	4,2			3.1		0.7
Application date			09.06.20	18.06.20	24.06.20	01.07.20	07.07.20	14.07.20	21.07.20	23.07.20	30.07.20	03.08.20	04.08.20	07.08.20	12.08.20
Water supply [L m^{-2}]	ZI-ZN	0	0	0	0	0	0	0	0	0	0	0	0	0	0
	SI-ZN	0	10	0	15	0	0	0	10	15	15	15	0	15	15
	DI-ZN	0	3.11	0	4.66	0	0	0	3.11	4.66	4.66	4.66	0	4.66	4.66
	ZI-N	0	0	0	0	0	0	0	0	0	0	0	0	0	0
	SI-N	0	10	0	15	0	0	0	10	15	15	15	0	15	15
	DI-N	0	3.11	0	4.66	0	0	0	3.11	4.66	4.66	4.66	0	4.66	4.66
	F	0	3.11	3.11	4.66	3.11	3.11	3.11	3.11	4.66	4.66	0	4.66	4.66	4.66
	F-ZC	0	3.11	3.11	4.66	3.11	3.11	3.11	3.11	4.66	4.66	0	4.66	4.66	4.66
Nitrogen supply [kg N ha^{-1}]	ZI-ZN	0	0	0	0	0	0	0	0	0	0	0	0	0	0
	SI-ZN	0	0	0	0	0	0	0	0	0	0	0	0	0	0
	DI-ZN	0	0	0	0	0	0	0	0	0	0	0	0	0	0
	ZI-N	0	75	0	0	0	45.33	0	0	0	0	0	30	0	0
	SI-N	0	75	0	0	0	45.33	0	0	0	0	0	30	0	0
	DI-N	0	75	0	0	0	45.33	0	0	0	0	0	30	0	0
	F	0	7.75	7.75	11.63	15.5	15.5	15.5	15.5	0	15.5	0	15.5	0	11.63
	F-ZC	0	7.75	7.75	11.63	15.5	15.5	15.5	15.5	0	15.5	0	15.5	0	11.63

Table S1: continued.

Sampling date		18.08.20	**25.08.20**	01.09.20	08.09.20	**15.09.20**
Air temperature [°C]		21.9	19.7	21.4	21.7	27.8
Soil temperature [°C]		23.2	18.5	14.4	14.5	18,0
Precipitation [mm]		0.1	5.1	17.1	3.5	0.0
Application date		19.08.20	27.08.20	03.09.20	09.09.20	harvest
Water supply [L m^{-2}]	ZI-ZN	0	0	0	0	
	SI-ZN	10	0	0	0	
	DI-ZN	3.11	0	0	0	
	ZI-N	0	0	0	0	
	DI-N	3.11	0	0	0	
	SI-N	10	0	0	0	
	F	3.11	3.11	3.11	3.11	
	F-ZC	3.11	3.11	3.11	3.11	
Nitrogen supply [kg N ha^{-1}]	ZI-ZN	0	0	0	0	
	SI-ZN	0	0	0	0	
	DI-ZN	0	0	0	0	
	ZI-N	0	0	0	0	
	SI-N	0	0	0	0	
	DI-N	0	0	0	0	
	F	7.75	3.88	3.88	3.88	
	F-ZC	7.75	3.88	3.88	3.88	

Table S2: Overview of different nitrogen (N) forms extracted from the Nmin sampling per sampling date for the investigated treatments ZI-ZN (zero irrigation without (zero) N application), ZI-N (zero irrigation with N application), SI-ZN (sprinkler irrigation without (zero) N application), SI-N (sprinkler irrigation with N application), DI-ZN (drip irrigation without (zero) N application), DI-N (drip irrigation with N application), F (fertigation) and F-ZC (fertigation without (zero) crops). Marked in bold are sampling dates for functional genes analysis. $NH4^+$= ammonia $NO2^-$= nitrite $NO3^-$ = nitrate

Treatment	**ZI-ZN**			**SI-ZN**			**DI-ZN**			**ZI-N**		
Sampling date	NH_4^+	NO_2^-	NO_3^-	NH_4^+	NO_2^-	NO_3^-	NH_4^+	NO_2^-	NO_3^-	NH_4^+	NO_2^-	NO_3^-
26.05.20	5.71	0.05	5.26	2.27	0.02	6.19	2.81	0.06	4.87	2.59	0.04	4.61
30.06.20	1.29	0.04	0.41	1.02	0.05	0.56	2.65	0.04	0.99	4.39	0.05	12.94
14.07.20	1.05	0.05	1.11	0.65	0.05	1.34	1.94	0.06	1.21	6.21	0.05	26.70
25.08.20	1.05	0.05	1.11	1.76	0.05	2.18	1.70	0.04	5.61	3.40	0.03	22.48
15.09.20	2.10	0.04	2.45	1.27	0.07	2.37	1.06	0.06	2.20	2.75	0.06	28.42

Treatment	**DI-N**			**SI-N**			**F**			**F-ZC**		
Sampling date	NH_4^+	NH_4^+	NO_2^-	NO_3^-	NH_4^+	NH_4^+	NO_2^-	NO_3^-	NH_4^+	NH_4^+	NO_2^-	NO_3^-
26.05.20	5.49	0.04	5.15	7.16	5.49	2.63	0.04	7.16	5.49	2.63	0.03	6.17
30.06.20	2.34	0.07	21.29	1.56	2.34	1.18	0.03	1.56	2.34	1.18	0.04	12.48
14.07.20	4.43	0.07	5.88	9.10	4.43	3.75	0.05	9.10	4.43	3.75	0.06	25.28
25.08.20	3.25	0.07	27.47	14.96	3.25	1.67	0.07	14.96	3.25	1.67	0.05	17.33
15.09.20	1.66	0.08	8.85	10.05	1.66	0.87	0.08	10.05	1.66	0.87	0.04	23.92

Table S3: Seasonal N_2O flux rates in µg N_2O-N m^{-2} h^{-1} given as median value of three field replicates. Factor of change in N_2O flux rates for the investigated treatment each compared to the untreated reference treatment zero irrigation without (zero) ZI-ZN. Abbreviations: ZI-N (zero irrigation with N application), SI-ZN (sprinkler irrigation without (zero) N application), SI-N (sprinkler irrigation with N application), DI-ZN (drip irrigation without (zero) N application), DI-N (drip irrigation with N application), F (fertigation) and F-ZC (fertigation without (zero) crops). Marked in bold are sampling dates for functional genes analysis.

	Seasonal N_2O flux rates per Treatment							
Sampling date	ZI-ZN	SI-ZN	DI-ZN	ZI-N	SI-N	DI-N	F	F-ZC
**26.05.20**	5.0	27.4	8.2	21.8	22.8	13.8	28.9	18.2
09.06.20	34.5	54.7	14.3	6.1	37.3	74.5	32.2	-11.0
16.06.20	25.6	40.0	30.7	38.1	28.0	43.3	34.8	-1.9
23.06.20	11.8	26.6	16.5	9.9	26.5	19.3	6.4	2.2
**30.06.20**	5.3	24.2	21.1	42.6	47.9	46.0	75.1	28.8
07.07.20	-9.7	9.2	-10.8	19.1	50.6	71.7	13.9	-1.3
**14.07.20**	22.8	32.8	8.7	11.5	52.1	80.4	34.7	43.0
21.07.20	-17.8	35.3	-8.1	50.2	12.5	9.7	28.4	17.7
28.07.20	-0.8	14.3	9.5	43.0	13.3	13.6	22.6	17.5
04.08.20	8.2	24.3	26.6	9.9	17.9	20.9	46.4	14.2
11.08.20	-5.5	23.3	18.1	14.8	27.7	13.8	7.8	12.9
18.08.20	-1.2	20.3	-2.9	10.7	19.3	19.5	12.1	0.7
**25.08.20**	6.5	3.3	30.1	14.2	3.8	5.8	9.4	4.5
01.09.20	24.8	12.5	10.3	-9.2	14.3	12.1	15.4	-5.4
08.09.20	15.6	9.1	-3.9	28.3	7.9	12.8	1.8	9.1
**15.09.20**	21.7	-1.6	-1.7	3.0	4.1	10.3	2.1	4.0
Seasonal median	7.3	23.7	9.9	14.5	21.1	16.5	19.0	6.8
Factor of change		3.2	1.4	2.0	2.9	2.3	2.6	0.9

Table S4: (a) Modell parameter (chi square and *p* values) of linear mixed effects models with `$N_2O$ flux´ as response variable and `week´ as explanatory variables. A separate model was fitted for each treatment: ZI-ZN (zero irrigation without (zero) nitrogen (N) fertilizer), SI-ZN (sprinkler irrigation without (zero) nitrogen (N) fertilizer), DI-ZN (drip irrigation without (zero) nitrogen (N) fertilizer), ZI-N (zero irrigation with nitrogen (N) fertilizer), SI-N (sprinkler irrigation with nitrogen (N) fertilizer), DI-N (drip irrigation with nitrogen (N) fertilizer), F (Fertigation), and F-ZC (Fertigation without crops). (b) Seasonal development of N_2O fluxes during the growing season for the three different treatments: ZI-ZN, SI-ZN, and DI-ZN shown are the median values per treatment and sampling date. Differences in lowercase letters (Group) indicate significant differences between sampling weeks (ANOVA followed by Tukey post-hoc-test). (c) Seasonal median values for each treatment with corresponding factor of change compared to reference treatment (ZI-ZN). Values with *, **, *** indicate significance at P < 0.05, 0.01, 0.001.

	Treatment	**ZI-ZN**				**SI-ZN**				**DI-ZN**			
(a)	ANOVA	*chi square*: 23.946		*p*: 0.0060*		*chi square*: 11.87		*p*: 0.6884		*chi square*: 32.164		*p*: <0.0061***	
(b)	Sampling date	Median	Group	Mean	SD	Median	Group	Mean	SD	Median	Group	Mean	SD
	26.05.20	5.02	ab	14.49	17.91	27.41	a	30.87	7.25	8.21	a	3.77	12.39
	09.06.20	34.53	b	43.04	23.68	54.66	a	46.55	15.33	14.32	a	19.77	20.95
	16.06.20	26.1	ab	37.56	20.72	39.97	a	32.31	14.75	30.69	a	52.54	38.28
	23.06.20	11.78	ab	13.09	6.46	26.63	a	17.1	23.07	16.54	a	18.26	4.71
	30.06.20	5.34	ab	10.59	13.86	13.95	a	20.81	28.33	21.10	a	23.18	7.99
	07.07.20	-9.74	ab	-7.69	32.15	9.16	a	13.8	22.16	1.64	a	-6.64	7.64
	14.07.20	22.75	ab	26.49	15.1	32.80	a	27.95	11.58	8.70	a	8.38	29.99
	21.07.20	-17.78	a	-14.12	17.06	35.26	a	32.83	7.91	-8.09	a	-6.63	23.35
	28.07.20	-0.82	ab	-3.76	9.06	14.30	a	11.07	7.62	9.54	a	5.01	16.7
	04.08.20	8.16	ab	1.6	28.91	24.32	a	40.69	71.12	26.59	a	27.11	7.95
	11.08.20	-5.45	ab	-4.49	11.54	23.25	a	43.91	63.53	18.06	a	15.18	6.01
	18.08.20	-1.25	ab	0.2	7.51	20.26	a	37.13	53.36	-2.91	a	24.68	54.56
	25.08.20	6.48	ab	16.82	20.41	3.26	a	0.3	33.03	30.08	a	29.3	16.41
	01.09.20	24.83	ab	33.55	28.44	12.54	a	10.93	12.51	10.34	a	10.27	1.96
	08.09.20	15.60	ab	15.6	26.75	9.15	a	-12.85	38.64	-3.91	a	-2.32	3.46
	15.09.20	21.74	ab	12.88	17.1	-1.64	a	12.11	23.82	-1.66	a	-2.1	1.22
(c)	Seasonal median	7.3				23.7				9.9			
	Factor of change					3.2				1.4			

Table S4: continued.

	Treatment	ZI-N				SI-N				DI-N			
(a)	ANOVA	*chi square*:	24.584	*p*:	0.0558	*chi square:*	*42.624*	*p*:	0.0018**	*chi square:*	24.584	*p*:	0.0558
(b)	Sampling date	Median	Group	Mean	*SD*	*Median*	*Group*	Mean	SD	Median	Group	Mean	SD
	26.05.20	21.83	a	12.31	22.56	*22.82*	abc	23.41	9.21	13.78	a	10.34	19.26
	09.06.20	6.07	a	21.54	41.85	*37.28*	abc	33.26	10.32	74.49	a	60.01	32.11
	16.06.20	38.13	a	39.82	6.03	*28.00*	abc	21.66	18.55	43.33	a	42.6	17.99
	23.06.20	9.88	a	10.74	10.07	*26.52*	abc	18.61	16.19	19.29	a	20.88	7.21
	30.06.20	42.62	a	51.23	16.57	*47.87*	bc	59.18	27.49	45.97	a	40.57	24.91
	07.07.20	-1.32	a	5.97	30.58	*50.65*	bc	56.45	17.26	71.65	a	51.82	40.87
	14.07.20	11.53	a	15.79	15.87	*52.05*	bc	50.19	13.69	80.40	a	77.33	36.1
	21.07.20	50.23	a	58.03	14.79	*12.53*	a	-0.08	25.41	9.68	a	13.96	37.98
	28.07.20	43.03	a	23.92	36.27	*13.30*	abc	13.09	2.57	13.60	a	11.02	15.69
	04.08.20	9.88	a	25.17	27.05	*17.91*	abc	37.83	35.19	20.89	a	28.67	54.02
	11.08.20	14.80	a	2.94	28.33	*27.74*	abc	27.43	6.57	13.78	a	13.28	3.78
	18.08.20	10.67	a	5.44	16.55	*19.30*	abc	26.75	12.93	16.57	a	-5.9	44.08
	25.08.20	14.21	a	18.27	15.77	3.77	ab	4.48	15.42	5.79	a	14.16	27.1
	01.09.20	-9.20	a	-3.09	27.06	14.33	abc	9.97	21.5	12.10	a	15.06	7.95
	08.09.20	28.29	a	16.24	31.23	7.90	abc	15.01	14.98	12.83	a	17.55	13.5
	15.09.20	3.03	a	-5.98	17.44	2.73	abc	3,49	1,68	10.28	a	17,27	19,03
(c)	Seasonal median	14.5				21.1				16.5			
	Factor of change	2.0				2.9				2.3			

Table S4: continued.

	Treatment	F				F-ZC			
(a)	ANOVA	*chi square*: 19.965		*p*: 0.1733		*chi square:* 10.876		*p*: 0.7613	
(b)	Sampling date	Median	Group	Mean	SD	Median	Group	Mean	SD
	26.05.20	28.93	a	20.66	29.41	18.18	a	21.44	17.33
	09.06.20	32.21	a	33.88	4.75	-10.98	a	3.87	49.55
	16.06.20	34.84	a	35.57	32.06	-1.92	a	7.25	16.72
	23.06.20	8.97	a	12.7	11.12	2.15	a	-3.09	10.41
	30.06.20	75.12	a	75.72	3.19	28.76	a	29.67	4.96
	07.07.20	13.93	a	18.46	15.45	-1.32	a	11.5	41.89
	14.07.20	34.71	a	30.71	10.89	30.68	a	34.72	18.87
	21.07.20	28.40	a	23.11	25.62	14.61	a	11.35	11.5
	28.07.20	22.60	a	44.03	54.12	17.49	a	18.84	24.37
	04.08.20	46.38	a	35.47	29.83	14.22	a	6.06	27.71
	11.08.20	7.80	a	11.04	7.18	12.91	a	19.52	13.74
	18.08.20	12.08	a	5.77	22.61	0.73	a	0.8	0.56
	25.08.20	9.41	a	21.6	24.06	4.49	a	7.06	33.32
	01.09.20	15.36	a	39.04	52.6	-5.37	a	-11.3	33.18
	08.09.20	1.79	a	7.22	29.77	9.13	a	14.16	12.13
	15.09.20	2.07	a	6.33	20.71	3.96	a	10.62	14.69
(c)	Seasonal median	19.0				6.8			
	Factor of change	2.6				0.6			

Table S5: Median values of the gene copy numbers per gram soil of the nitrogen cycle for the investigated treatments ZI-ZN (zero irrigation without (zero) N application), ZI-N (zero irrigation with N application), SI-ZN (sprinkler irrigation without (zero) N application), SI-N (sprinkler irrigation with N application), DI-ZN (drip irrigation without (zero) N application), DI-N (drip irrigation with N application), F (fertigation) and F-ZC (fertigation without (zero) crops) of the ridge (0-10cm). Modell parameter (*F* and *p* values) of generalized mixed effects models with `gene copy numbers´ as response variable and `week´ as explanatory variables. A separate model was fitted for each treatment. Differences in lowercase letters indicate significant differences between sampling weeks (ANOVAs followed by Tukey post-hoc tests, $P < 0.05$) under consideration that sampling dates were uneven in time space. *amo*A = gene encoding ammonium monooxygenase, *nxr*B = gene encoding nitrite oxidoreductase, *na*rG = gene encoding nitrate reductase, *nir*K/*nir*S = gene encoding nitrite reductase and *nos*Z = encoding nitrous oxide (N_2O) reductase.

Sampling date*	26.05.20	30.06.20	14.07.20	25.08.20	15.09.20	ANOVA	
						F	ρ
***amo*A**							
ZI-ZN	9.67 x10^4	2.34 x10^4	3.50 x10^4	6.29 x10^4	4.40 x10^4	6.798	0.0065
SI-ZN	1.30x10^5b	3.20 x10^4a	5.21x10^4a	5.52 x10^4a	4.39x10^4a	27.841	<0.05
DI-ZN	4.03 x10^4	3.41 x10^4	6.77 x10^4	2.93 x10^4	2.86 x10^4	3.327	0.056
ZI-N	6.32 x10^4ab	3.36x10^4 a	7.45x10^4b	5.55x10^4ab	3.48x10^4ab	4.381	<0.05
SI-N	4.42 x10^4a	3.37x10^4a	8.24x10^4b	4.21x10^4ab	3.94x10^4a	5.576	<0.05
DI-N	4.02 x10^4ab	2.87x10^4ab	1.05x10^4b	3.02x10^4a	3.11x10^4ab	9.842	<0.05
F	1.66x10^5b	3.26 x10^4ab	6.03x10^4ab	4.96 x10^4a	5.61x10^4a	32.316	<0.05
F-ZC	1.00x10^5b	2.34 x10^4a	4.51 x10^4a	5.21 x10^4a	3.20 x10^4a	10.187	<0.05
***nxr*B**							
ZI-ZN	2.67 x10^5	3.64 x10^5	3.13 x10^5a	2.79 x10^5	2.15 x10^5	1.416	0.979
SI-ZN	2.47 x10^5	2.92 x10^5	2.39 x10^5	3.02 x10^5	3.00 x10^5	1.561	0.258
DI-ZN	2.07 x10^5	3.05 x10^5	3.34 x10^5	5.02 x10^5	3.34 x10^5	2.302	0.130
ZI-N	2.47 x10^5	2.14x10^5	1.67 x10^5	2.68x10^5	2.95 x10^5	2.650	0.096
SI-N	2.39 x10^5a	2.58x10^5ab	3.81 x10^5 ab	5.05x10^5b	4.50x10^5ab	3.962	<0.05
DI-N	2.45x10^5	3.10 x10^5	2.80 x10^5	3.92 x10^5	3.91 x10^5	1.525	0.387
F	2.94x10^5	3.58 x10^5	3.11 x10^5	3.68 x10^5	3.68 x10^5	2.981	0.073
F-ZC	3.54x10^5	2.72 x10^5	2.80x10^5	2.96 x10^5	2.30 x10^5	1.012	0.446
narG							
ZI-ZN	3.12 x10^7c	2.22 x10^7b	2.25 x10^7bc	1.35 x10^7a	1.16 x10^7a	33.486	<0.05
SI-ZN	2.78 x10^7a	2.25 x10^7c	1.66 x10^7b	1.24 x10^7a	2.02 x10^7bc	32.190	<0.05
DI-ZN	1.73 x10^7ab	3.32 x10^7b	3.14 x10^7ab	1.95 x10^7a	1.13 x10^7a	12.200	<0.05
ZI-N	1.82 x10^7ab	2.58 x10^7b	2.22 x10^7ab	1.67 x10^7a	1.25 x10^7a	5.440	<0.05
SI-N	1.62 x10^7a	2.83 x10b^7	3.30 x10^7b	1.90 x10^7a	1.46 x10^7a	17.016	<0.05
DI-N	1.67 x10^7a	2.75 x10^7b	2.65 x10^7b	1.69 x10^7a	1.43 x10^7a	10.928	<0.05
F	3.39 x10^7d	2.33 x10^7c	2.00 x10^7b	1.26 x10^7a	1.98 x10^7bc	47.064	<0.05
F-ZC	3.58 x10^7b	1.88 x10^7a	1.73 x10^7a	1.29 x10^7a	1.20 x10^7a	12.419	<0.05

Table S5: continued.

Sampling date*	26.05.20	30.06.20	14.07.20	25.08.20	15.09.20	ANOVA	
						F	ρ
nirK							
ZI-ZN	2.33 x10^6	2.08x10^6	1.28x10^6	2.15x10^6	2.15x10^6	4.327	0.028
SI-ZN	7.28 x10^{5}a	2.06 x10^{6}c	9.04 x10^{5}a	2.28x10^{6}bc	1.48x10^{6}ab	12.256	<0.05
DI-ZN	1.19 x10^6	2.09x10^6	1.27x10^6	2.05x10^6	1.96x10^6	3.753	0.041
ZI-N	1.31 x10^6	2.04x10^5	6.14x10^5	1.16x10^6	2.17x10^6	4.327	0.041
SI-N	1.29 x10^{6}a	1.74 x10^{6}b	1.42x10^{6}a	2.07x10^{6}b	1.59x10^{6}a	17.323	<0.05
DI-N	1.22x10^{6}ac	2.32x10^{6}bd	1.02x10^{6}ab	1.92x10^{6}cd	1.31x10^{6}abcd	22.651	<0.05
F	1.37x10^{6}a	2.01x10^{6}c	1.05x10^{6}a	2.38x10^{6}bc	1.68x10^{6}ab	23.697	<0.05
F-ZC	1.65x10^{6}ab	1.94x10^{6}b	7.94x10^{5}a	2.18x10^{6}b	1.06x10^{6}ab	5.822	<0.05
nirS							
ZI-ZN	1.75x10^{5}ab	1.34x10^{5}a	2.18x10^{5}ab	2.33x10^{5}b	2.47x10^{5}b	4.667	<0.05
SI-ZN	1.87x10^{5}ab	1.43x10^{5}a	1.40x10^{5}a	2.12x10^{5a}b	2.58x10^{5}b	8.793	<0.05
DI-ZN	1.50x10^5	2.83x10^5	2.18x10^5	2.36x10^5	2.19x10^5	2.303	0.130
ZI-N	1.97x10^{5}ab	2.23x10^{5}b	1.24x10^5 a	1.71x10^{5}ab	2.26x10^6 ab	4.259	<0.05
SI-N	1.61x10^{5}a	2.56x10^{5}ab	2.35x10^5 ab	2.25x10^{5}ab	3.19x10^6 b	5.233	<0.05
DI-N	1.92x10^5	2.33x10^5	1.72x10^5	1.92x10^5	2.43x10^5	3.403	0.053
F	2.60x10^{5}ab	1.81x10^{5}a	1.87x10^5 a	2.23x10^{5}a	3.32x10^6 b	8.030	<0.05
F-ZC	2.49x10^{5}b	1.01x10^{5}a	1.69x10^5 ab	2.01x10^{5}ab	1.93x10^5 ab	3.408	<0.05
nosZ							
ZI-ZN	1.20x10^{5}a	1.94x10^{5}b	1.89x10^{5}b	1.07x10^{5}ab	1.19x10^{5}ab	4.956	<0.05
SI-ZN	8.41x10^{4}a	6.38x10^{4}a	1.33x10^{4}b	8.46x10^{4}a	1.67x10^{5}b	13.263	<0.05
DI-ZN	2.66x10^{5}a	2.42x10^{5}a	1.47x10^{5}a	1.67x10^{5}b	1.20x10^{5}a	48.17	<0.05
ZI-N	1.63 x10^{5}a	1.83x10^5 a	9.19x10^4 a	1.08x10^6 b	1.30x10^{5}a	37.702	<0.05
SI-N	2.91 x10^{5}a	2.07 x10^5 a	1.42x10^5 a	1.61x10^6 b	1.65x10^{5}a	41.078	<0.05
DI-N	4.53x10^{5}a	2.05x10^5 a	1.18x10^5 a	1.20x10^6 b	1.66x10^{5}a	100.329	<0.05
F	1.08x10^{5}a	8.15x10^5 a	1.65x10^5 a	1.03x10^6 b	1.76x10^{5}a	49.16	<0.05
F-ZC	1.08x10^5	9.80x10^4	1.65x10^5	9.80x10^5	1.33x10^5	2.650	0.096

Table S6: Pearson's correlations for the detection of time-independent relationships among the investigated gene copy numbers per gram soil and between the investigated genes and the main environmental factors for treatment ZI-ZN (zero irrigation without (zero) N application) of the ridge (0-10cm). *amo*A = gene encoding ammonium monooxygenase, *nxr*B = gene encoding nitrite oxidoreductase, *nar*G = gene encoding nitrate reductase, *nir*K/*nir*S = gene encoding nitrite reductase and *nos*Z = gene encoding nitrous oxide (N_2O) reductase, N_2O = nitrous oxide, WFPS = water filled pore space, Temp = soil temperature, NH_4^+ = ammonium, NO_2^- = nitrite, NO_3^- = nitrate. Values with *, **, *** indicate significance at P < 0.05, 0.01, 0.001.

**ZI-ZN**	*amo*A	*nxr*B	*nar*G	*nir*K	*nir*S	*nos*Z	N_2O	WFPS	Temp	NH_4^+	NO_2^-	NO_3^-
*amo*A	-											
*nxr*B	-0.48	-										
*nar*G	0.43	0.39	-									
*nir*K	0.57	0.28	0.46	-								
*nir*S	0.09	-0.76	-0.68	-0.58	-							
*nos*Z	-0.72	0.81	0.31	-0.27	-0,55	-						
N_2O	-0.42	-0.37	-0.39	-0.98	0.40	0.18	-					
WFPS	0.11	0.27	0.76	0.22	-0.74	0.39	-0.22	-				
Temp	-0.55	-0.16	-0.94	-0.51	0.65	-0.09	0.44	-0.86	-			
NH_4	0.93	-0.22	0.70	0.68	-0.27	-0.46	-0.56	0.44	-0.82	-		
NO_2^-	0.02	-0.78	-0.33	-0.59	0.49	-0.33	0.61	0.13	0.08	-0.08	-	
NO_3^-	0.86	-0.80	0.19	0.23	0.29	-0.80	-0.10	0.17	-0.43	0.76	0.52	-

Table S7: Pearson's correlations for the detection of time-independent relationships among the investigated gene copy numbers per gram soil and between the investigated genes and the main environmental factors for treatment SI-ZN (sprinkler irrigation without (zero) N application) of the ridge (0-10cm). *amo*A = gene encoding ammonium monooxygenase, *nxr*B = gene encoding nitrite oxidoreductase, *nar*G = gene encoding nitrate reductase, *nir*K/*nir*S = gene encoding nitrite reductase and *nos*Z = gene encoding nitrous oxide (N_2O) reductase, N_2O = nitrous oxide, WFPS = water filled pore space, Temp = soil temperature, NH_4^+ = ammonium, NO_2^- = nitrite, NO_3^- = nitrate. Values with *, **, *** indicate significance at P < 0.05, 0.01, 0.001.

**SI-ZN**	*amo*A	*nxr*B	*nar*G	*nir*K	*nir*S	*nos*Z	N_2O	WFPS	Temp	NH_4^+	NO_2^-	NO_3^-
*amo*A	-											
*nxr*B	-0.58	-										
*nar*G	0.59	-0.36	-									
*nir*K	-0.64	0.88	-0.53	-								
*nir*S	0.04	0.56	-0.10	0.16	-							
*nos*Z	-0.22	-0.02	-0.19	-0.35	0.52	-						
N_2O	0.32	-0.84	0.41	-0.57	-0.90	-0.37	-					
WFPS	0.22	-0.10	0.77	-0.02	-0.46	-0.66	0.49	-				
Temp	-0.84	0.58	-0.87	0.58	0.30	0.46	-0.60	-0.70	-			
NH_4	0.79	0.03	0.37	-0.08	0.42	-0.36	-0.21	0.16	-0.56	-		
NO_2^-	-0.80	0.63	-0.55	0.44	0.50	0.67	-0.69	-0.55	0.89	-0.55	-	
NO_3^-	0.97	-0.41	0.60	-0.59	0.29	-0.09	0.10	0.15	-0.75	0.86	-0.65	-

Table S8: Pearson's correlations for the detection of time-independent relationships among the investigated gene copy numbers per gram soil and between the investigated genes and the main environmental factors for treatment DI-ZN (drip irrigation without (zero) N application) of the ridge (0-10cm). *amo*A = gene encoding ammonium monooxygenase, *nxr*B = gene encoding nitrite oxidoreductase, *nar*G = gene encoding nitrate reductase, *nir*K/*nir*S = gene encoding nitrite reductase and *nos*Z = gene encoding nitrous oxide (N_2O) reductase, N_2O = nitrous oxide, WFPS = water filled pore space, Temp = soil temperature, NH_4^+ = ammonium, NO_2^- = nitrite, NO_3^- = nitrate. Values with *, **, *** indicate significance at P < 0.05, 0.01, 0.001.

**DI-ZN**	*amo*A	*nxr*B	*nar*G	*nir*K	*nir*S	*nos*Z	N_2O	WFPS	Temp	NH_4^+	NO_2^-	NO_3^-
*amo*A	-											
*nxr*B	-0.24	-										
*nar*G	0.56	-0.04	-									
*nir*K	-0.73	0.59	-0.06	-								
*nir*S	-0.20	0.47	0.55	0.79	-							
*nos*Z	-0.39	0.83	-0.15	0.41	0.15	-						
N_2O	-0.23	0.61	0.40	0.44	0.48	0.80	-					
WFPS	-0.22	-0.67	0.11	-0.20	-0.23	-0.22	0.11	-				
Temp	-0.08	0.87	0.05	0.61	0.63	0.45	0.28	-0.87	-			
NH_4	0.31	-0.47	0.81	-0.18	0.26	-0.30	0.31	0.67	-0.49	-		
NO_2^-	-0.23	-0.20	-0.93	-0.28	-0.75	-0.12	-0.66	-0.13	-0.20	-0.75	-	
NO_3^-	-0.40	0.28	-0.61	-0.09	-0.55	0.70	0.31	0.12	-0.16	-0.42	0.48	-

Table S9: Pearson's correlations for the detection of time-independent relationships among the investigated gene copy numbers per gram soil and between the investigated genes and the main environmental factors for treatment ZI-N (zero irrigation with N application) of the ridge (0-10cm). *amo*A = gene encoding ammonium monooxygenase, *nxr*B = gene encoding nitrite oxidoreductase, *nar*G = gene encoding nitrate reductase, *nir*K/*nir*S = gene encoding nitrite reductase and *nos*Z = gene encoding nitrous oxide (N_2O) reductase, N_2O = nitrous oxide, WFPS = water filled pore space, Temp = soil temperature, NH_4^+ = ammonium, NO_2^- = nitrite, NO_3^- = nitrate. Values with *, **, *** indicate significance at P < 0.05, 0.01, 0.001.

**ZI-N**	*amo*A	*nxr*B	*nar*G	*nir*K	*nir*S	*nos*Z	N_2O	WFPS	Temp	NH_4^+	NO_2^-	NO_3^-
*amo*A	-											
*nxr*B	-0.45	-										
*nar*G	0.12	-0.92	-									
*nir*K	-0.97	0.50	-0.19	-								
*nir*S	-0.89	0.53	-0.21	0.96	-							
*nos*Z	0.05	0.34	-0.24	-0.21	-0.16	-						
N_2O	-0.29	-0.53	0.81	0.25	0.32	-0.10	-					
WFPS	-0.33	-0.36	0.53	0.45	0.53	-0.66	0.74	-				
Temp	-0.22	0.16	-0.17	0.02	-0.21	0.37	-0.39	-0.68	-			
NH_4	0.44	-0.84	0.67	-0.56	-0.71	-0.22	0.13	-0.09	0.36	-		
NO_2^-	-0.28	0.03	-0.14	0.36	0.19	-0.79	-0.31	0.22	0.20	0.18	-	
NO_3^-	-0.04	0.21	-0.36	-0.09	-0.32	0.13	-0.68	-0.72	0.91	0.35	0.44	-

Table S10: Pearson's correlations for the detection of time-independent relationships among the investigated gene copy numbers per gram soil and between the investigated genes and the main environmental factors for treatment SI-N (sprinkler irrigation with N application) of the ridge (0-10cm). *amo*A = gene encoding ammonium monooxygenase, *nxr*B = gene encoding nitrite oxidoreductase, *nar*G = gene encoding nitrate reductase, *nir*K/*nir*S = gene encoding nitrite reductase and *nos*Z = gene encoding nitrous oxide (N_2O) reductase, N_2O = nitrous oxide, WFPS = water filled pore space, Temp = soil temperature, NH_4^+ = ammonium, NO_2^- = nitrite, NO_3^- = nitrate. Values with *, **, *** indicate significance at P < 0.05, 0.01, 0.001.

**SI-N**	*amo*A	*nxr*B	*nar*G	*nir*K	*nir*S	*nos*Z	N_2O	WFPS	Temp	NH_4^+	NO_2^-	NO_3^-
*amo*A	-											
*nxr*B	0.11	-										
*nar*G	0.62	-0.22	-									
*nir*K	-0.41	0.62	-0.06	-								
*nir*S	-0.16	0.48	-0.02	0.29	-							
*nos*Z	-0.22	0.61	-0.27	0.81	-0.20	-						
N_2O	0.51	-0.60	0.90	-0.39	-0.18	-0.55	-					
WFPS	-0.49	-0.89	0.03	-0.22	-0.45	-0.27	0.36	-				
Temp	0.16	0.83	0.21	0.64	0.75	0.33	-0.17	-0.75	-			
NH_4	0.89	-0.19	0.52	-0.54	-0.58	-0.17	0.53	-0.16	-0.26	-		
NO_2^-	-0.02	0.90	-0.48	0.36	0.62	0.33	-0.74	-0.85	0.68	-0.34	-	
NO_3^-	0.19	0.86	-0.39	0.41	0.02	0.71	-0.69	-0.79	0.46	0.09	0.76	-

Table S11: Pearson's correlations for the detection of time-independent relationships among the investigated gene copy numbers per gram soil and between the investigated genes and the main environmental factors for treatment DI-N (drip irrigation with N application) of the ridge (0-10cm). *amo*A = gene encoding ammonium monooxygenase, *nxr*B = gene encoding nitrite oxidoreductase, *nar*G = gene encoding nitrate reductase, *nir*K/*nir*S = gene encoding nitrite reductase and *nos*Z = gene encoding nitrous oxide (N_2O) reductase, N_2O = nitrous oxide, WFPS = water filled pore space, Temp = soil temperature, NH_4^+ = ammonium, NO_2^- = nitrite, NO_3^- = nitrate. Values with *, **, *** indicate significance at P < 0.05, 0.01, 0.001.

**DI-N**	*amo*A	*nxr*B	*nar*G	*nir*K	*nir*S	*nos*Z	N_2O	WFPS	Temp	NH_4^+	NO_2^-	NO_3^-
*amo*A	-											
*nxr*B	-0.46	-										
*nar*G	0.50	-0.45	-									
*nir*K	-0.66	0.28	0.25	-								
*nir*S	-0.69	047	-0.19	0.44	-							
*nos*Z	-0.39	0.42	-0.42	0.35	-0.29	-						
N_2O	0.83	-0.49	0.88	-0.23	-0.38	-0.58	-					
WFPS	-0.37	-0.60	0.19	0.33	0.32	-0.35	-0.06	-				
Temp	0.07	0.83	0.00	0.09	0.18	0.18	0.07	-0.82	-			
NH_4	-0.12	-0.58	-0.31	-0.12	-0.47	0.36	-0.34	0.44	-0.82	-		
NO_2^-	0.22	0.73	-0.02	-0.16	0.21	-0.08	0.17	-0.81	0.95	-0.87	-	
NO_3^-	0.37	0.40	0.01	-0.09	-0.60	0.61	0.12	-0.87	0.63	-0.17	0.50	-

Table S12: Pearson's correlations for the detection of time-independent relationships among the investigated gene copy numbers per gram soil and between the investigated genes and the main environmental factors for treatment F (fertigation) of the ridge (0-10cm). *amo*A = gene encoding ammonium monooxygenase, *nxr*B = gene encoding nitrite oxidoreductase, *nar*G = gene encoding nitrate reductase, *nir*K/*nir*S = gene encoding nitrite reductase and *nos*Z = gene encoding nitrous oxide (N_2O) reductase, N_2O = nitrous oxide, WFPS = water filled pore space, Temp = soil temperature, NH_4^+ = ammonium, NO_2^- = nitrite, NO_3^- = nitrate. Values with *, **, *** indicate significance at P < 0.05, 0.01, 0.001.

**F**	*amo*A	*nxr*B	*nar*G	*nir*K	*nir*S	*nos*Z	N_2O	WFPS	Temp	NH_4^+	NO_2^-	NO_3^-
*amo*A	-											
*nxr*B	-0.78	-										
*nar*G	0.81	-0.73	-									
*nir*K	-0.54	0.70	-0.31	-								
*nir*S	0.29	0.19	0.12	-0.29	-							
*nos*Z	-0.07	-0.06	-0.19	-0.73	0.50	-						
N_2O	-0.17	-0.16	0.33	0.44	-0.73	-0.58	-					
WFPS	0.37	-0.65	0.80	-0.21	-0.29	-0.12	0.71	-				
Temp	-0.85	0.76	-0.98	0.25	0.00	0.34	-0.36	-0.75	-			
NH_4	0.64	-0.72	0.85	-0.04	-0.38	-0.57	0.64	0.79	-0.92	-		
NO_2^-	-0.34	0.52	-0.63	-0.23	0.64	0.73	-0.83	-0.72	0.72	-0.93	-	
NO_3^-	-0.20	-0.03	-0.64	-0.34	-0.26	0.32	-0.46	-0.57	0.56	-0.48	0.41	-

Table S13: Pearson's correlations for the detection of time-independent relationships among the investigated gene copy numbers per gram soil and between the investigated genes and the main environmental factors for treatment F-ZC (fertigation without (zero) crops) of the ridge (0-10cm). *amo*A = gene encoding ammonium monooxygenase, *nxr*B = gene encoding nitrite oxidoreductase, *nar*G = gene encoding nitrate reductase, *nir*K/*nir*S = gene encoding nitrite reductase and *nos*Z = gene encoding nitrous oxide (N_2O) reductase, N_2O = nitrous oxide, WFPS = water filled pore space, Temp = soil temperature, NH_4^+ = ammonium, NO_2^- = nitrite, NO_3^- = nitrate. Values with *, **, *** indicate significance at P < 0.05, 0.01, 0.001.

**F-ZC**	*amo*A	*nxr*B	*nar*G	*nir*K	*nir*S	*nos*Z	N_2O	WFPS	Temp	NH_4^+	NO_2^-	NO_3^-
*amo*A	-											
*nxr*B	0.90	-										
*nar*G	0.84	0.86	-									
*nir*K	0.12	0.38	0.12	-								
*nir*S	0.84	0.55	0.46	-0.07	-							
*nos*Z	-0.15	-0.33	-0.21	-0.95	0.02	-						
N_2O	-0.09	0.12	0.21	-0.41	-0.44	0.52	-					
WFPS	0.09	0.28	0.51	-0.32	-0.35	0.33	0.92	-				
Temp	-0.82	-0.73	-0.96	0.09	-0.55	0.05	-0.14	-0.45	-			
NH_4	0.92	0.84	0.71	0.44	0.81	-0.48	-0.41	-0.21	-0.67	-		
NO_2^-	-0.35	-0.35	-0.61	-0.45	-0.12	0.68	0.32	-0.06	0.61	-0.50	-	
NO_3^-	-0.62	-0.77	-0.80	-0.66	-0.22	0.74	0.10	-0.21	0.67	-0.72	0.83	-

Table S14: Time dependent mixed effect model with \`$N_2O$ fluxes´ as response variable and \`week´ and \`gene copy numbers per gram soil´ and their interactions as variables. Model includes correction exponent due to the uneven time spacing between sampling dates. Correlation coefficients derived from two-way ANOVA for each gene for each treatment are shown. *amo*A = gene encoding ammonium monooxygenase, *nxr*B = gene encoding nitrite oxidoreductase, *nar*G = gene encoding nitrate reductase, *nir*K/*nir*S = gene encoding nitrite reductase and *nos*Z = gene encoding nitrous oxide (N_2O) reductase. Values with *, **, *** indicate significance at P < 0.05, 0.01, 0.001.

Treatment	**ZI-ZN (**zero irrigation without (zero) N application)											
Gene	*amo*A		*nxr*B		*nar*G		*nir*K		*nir*S		*nos*Z	
	Chisq	*p*	Chisq	*p*	Chisq	*p*	Chisq	*p*	Chisq	*p*	Chisq	*p*
Week	26.27	0.0009***	41.58	1.6 e-06***	14.3	0.074	40.91	2.17 e-06***	39.48	4 -06***	43.52	6.99 e-7***
Gene	0.144	0.995	3.74	0.053	1.25	0.263	3.1	0.078	0.31	0.577	3.48	0.062
Interaction	3.99	0.858	7.94	0.439	7.67	0.466	6.56	0.584	10.09	0.258	14.96	0.059
Treatment	**SI-ZN** (sprinkler irrigation without (zero) N application)											
Gene	*amo*A		*nxr*B		*nar*G		*nir*K		*nir*S		*nos*Z	
	Chisq	*p*	Chisq	*p*	Chisq	*p*	Chisq	*p*	Chisq	*p*	Chisq	*p*
Week	40.14	3.01 e-06***	34.56	3.21e-05***	34.72	3.0 e-05***	45.9	2.49 e-07***	22.14	0.005**	75.64	3.68e-13***
Gene	0.05	0.800	0.05	0.830	0.01	0.919	2.87	0.090	0.22	0.636	5.14	0.023*
Interaction	9.61	0.293	8.2	0.414	7.94	0.540	8.0	0.433	6.58	0.583	18.20	0.019*
Treatment	**DI-ZN** (drip irrigation without (zero) N application)											
Gene	*amo*A		*nxr*B		*nar*G		*nir*K		*nir*S		*nos*Z	
	Chisq	*p*	Chisq	*p*	Chisq	*p*	Chisq	*p*	Chisq	*p*	Chisq	*p*
Week	25.06	0.0015**	124.68	<2.2e-16***	43.15	8.22e-07***	64.1	7.28e-11***	36.3	1.55e-05***	68.85	8.3e-12***
Gene	0.55	0.458	0.001	0.921	0.0028	0.958	1.1	0.293	1.66	0.198	0.2	0.66
Interaction	2.15	0.976	743.56	6.Z-07***	8.68	0.370	26.76	0.0008***	17.28	0.03*	32.74	6.86e-05***

Table S14: continued.

Treatment	**ZI-N** (zero irrigation with N application)											
Gene	*amo*A		*nxr*B		*nar*G		*nir*K		*nir*S		*nos*Z	
	Chisq	*p*	Chisq	*p*	Chisq	*p*	Chisq	*p*	Chisq	*p*	Chisq	*p*
Week	40.14	3.01 e-06***	34.56	3.21e-05***	34.72	3.0 e-05***	45.9	2.49 e-07***	22.14	0.005**	75.64	3.68e-13***
Gene	0.05	0.800	0.05	0.830	0.01	0.919	2.87	0.090	0.22	0.636	5.14	0.023*
Interaction	9.61	0.293	8.2	0.414	7.94	0.540	8.0	0.433	6.58	0.583	18.20	0.019*
Treatment	**SI-N** (sprinkler irrigation with N application)											
Gene	*amo*A		*nxr*B		*nar*G		*nir*K		*nir*S		*nos*Z	
	Chisq	*p*	Chisq	*p*	Chisq	*p*	Chisq	*p*	Chisq	*p*	Chisq	*p*
Week	25.06	0.0015**	124.68	<2.2e-16***	43.15	8.22e-07***	64.1	7.28e-11***	36.3	1.55e-05***	68.85	8.3e-12***
Gene	0.55	0.458	0.001	0.921	0.0028	0.958	1.1	0.293	1.66	0.198	0.2	0.66
Interaction	2.15	0.976	743.56	6.87e-07***	8.68	0.370	26.76	0.0008***	17.28	0.03*	32.74	6.86e-05***
Treatment	**DI-N** (drip irrigation with N application)											
Gene	*amo*A		*nxr*B		*nar*G		*nir*K		*nir*S		*nos*Z	
	Chisq	*p*	Chisq	*p*	Chisq	*p*	Chisq	*p*	Chisq	*p*	Chisq	*p*
Week	26.27	0.0009***	41.58	1.6 e-06***	14.3	0.074	40.91	2.17 e-06***	39.48	4 -06***	43.52	6.99 e-7***
Gene	0.144	0.995	3.74	0.053	1.25	0.263	3.1	0.078	0.31	0.577	3.48	0.062
Interaction	3.99	0.858	7.94	0.439	7.67	0.466	6.56	0.584	10.09	0.258	14.96	0.059

Table S14: continued.

Treatment	**F** (fertigation)											
Gene	*amo*A		*nxr*B		*nar*G		*nir*K		*nir*S		*nos*Z	
	Chisq	*p*	Chisq	*p*	Chisq	*p*	Chisq	*p*	Chisq	*p*	Chisq	*p*
Week	26.27	0.0009***	41.58	1.6 e-06***	14.3	0.074	40.91	2.17 e-06***	39.48	4 -06***	43.52	6.99 e-7***
Gene	0.144	0.995	3.74	0.053	1.25	0.263	3.1	0.078	0.31	0.577	3.48	0.062
Interaction	3.99	0.858	7.94	0.439	7.67	0.466	6.56	0.584	10.09	0.258	14.96	0.059
Treatment	**F-ZC** (fertigation without (zero) crops)											
Gene	*amo*A		*nxr*B		*nar*G		*nir*K		*nir*S		*nos*Z	
	Chisq	*p*	Chisq	*p*	Chisq	*p*	Chisq	*p*	Chisq	*p*	Chisq	*p*
Week	40.14	3.01 e-06***	34.56	3.21e-05***	34.72	3.0 e-05***	45.9	2.49 e-07***	22.14	0.005**	75.64	3.68e-13***
Gene	0.05	0.800	0.05	0.830	0.01	0.919	2.87	0.090	0.22	0.636	5.14	0.023*
Interaction	9.61	0.293	8.2	0.414	7.94	0.540	8.0	0.433	6.58	0.583	18.20	0.019*
